# Treatment use in prognostic model research: a systematic review of cardiovascular prognostic studies

**DOI:** 10.1186/s41512-017-0015-0

**Published:** 2017-09-26

**Authors:** Romin Pajouheshnia, Johanna A. A. G. Damen, Rolf H. H. Groenwold, Karel G. M. Moons, Linda M. Peelen

**Affiliations:** 1 Julius Center for Health Sciences and Primary Care, University Medical Center Utrecht, Huispost Str.6.131, PO Box 85500, 3508 GA Utrecht, The Netherlands; 2 Cochrane Netherlands, University Medical Center Utrecht, Huispost Str.6.131, PO Box 85500, 3508 GA Utrecht, The Netherlands

## Abstract

**Background:**

Ignoring treatments in prognostic model development or validation can affect the accuracy and transportability of models. We aim to quantify the extent to which the effects of treatment have been addressed in existing prognostic model research and provide recommendations for the handling and reporting of treatment use in future studies.

**Methods:**

We first describe how and when the use of treatments by individuals in a prognostic study can influence the development or validation of a prognostic model. We subsequently conducted a systematic review of the handling and reporting of treatment use in prognostic model studies in cardiovascular medicine. Data on treatment use (e.g. medications, surgeries, lifestyle interventions), the timing of their use, and the handling of such treatment use in the analyses were extracted and summarised.

**Results:**

Three hundred two articles were included in the review. Treatment use was not mentioned in 91 (30%) articles. One hundred forty-six (48%) reported specific information about treatment use in their studies; 78 (26%) provided information about multiple treatments. Three articles (1%) reported changes in medication use (“treatment drop-in”) during follow-up. Seventy-nine articles (26%) excluded treated individuals from their analysis, 80 articles (26%) modelled treatment as an outcome, and of the 155 articles that developed a model, 86 (55%) modelled treatment use, almost exclusively at baseline, as a predictor.

**Conclusions:**

The use of treatments has been partly considered by the majority of CVD prognostic model studies. Detailed accounts including, for example, information on treatment drop-in were rare. Where relevant, the use of treatments should be considered in the analysis of prognostic model studies, particularly when a prognostic model is designed to guide the use of certain treatments and these treatments have been used by the study participants. Future prognostic model studies should clearly report the use of treatments by study participants and consider the potential impact of treatment use on the study findings.

**Electronic supplementary material:**

The online version of this article (10.1186/s41512-017-0015-0) contains supplementary material, which is available to authorized users.

## Background

An important part of prognostic research is the development and validation of prognostic models or risk scores. These models can be used to make individualised predictions of a person’s absolute risk of developing a specific health outcome [1, 2] and can, for example, be used to inform different aspects of clinical decision-making. A notable example of this is in cardiovascular medicine: if a patient’s risk of a cardiovascular event is predicted to be above a specific probability threshold, lifestyle changes are recommended, with or without initiation of preventative medication [3–5].

Concerns have been raised that the use of treatments, such as pharmacological therapy or diet and lifestyle-related interventions, may have an unwanted impact when patient data (e.g. from a cohort or registry) is used to develop or validate a prognostic model [6–8]. In order to develop or validate prognostic models that predict an individual’s probability of developing an outcome in the absence of a certain treatment (i.e. their untreated health course), one should ideally include people who have not received that treatment before or during follow-up [1, 6]. In practice, however, such prognostic models are often derived from or validated in data sets where a proportion of the individuals has received that specific treatment. If, for example, treatments were administered in a study according to individuals’ predicted risks (either implicitly or explicitly), a model developed using this data will likely underestimate the risk of the predicted outcome in the absence of treatment and could thus lead to under-treatment when such a model is used in future individuals [8, 9].

In this manuscript, we aim to provide insight into the problems that arise when treatment use is ignored when developing or validating a prognostic model. First, we elaborate on how and when treatment use could negatively impact prognostic modelling. Following this, we provide evidence of the scale of this issue in published studies by means of a systematic literature review of the reporting and handling of treatment use in cardiovascular prognostic model research. We conclude with suggestions for the handling and reporting of treatment use in prognostic model research.

## Methods

### What do we mean by “treatment” and when is it a problem?

**Fig. 1:**
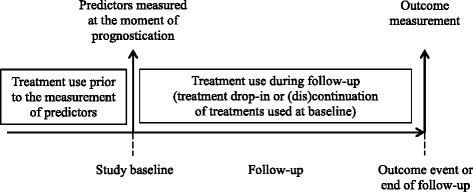
The timing of treatment use in a prognostic study

Herein, we use “treatment” to refer to any intervention, medical (e.g. medication, surgery, therapy) or non-medical (e.g. quit smoking or do more exercise), undertaken by an individual that lowers their risk of a certain outcome. We also include in this definition modifications that an individual makes to their behaviour or lifestyle that reduce their risk of a specific outcome. We propose two categories of treatment: “guided” and “background”. The term “guided treatments” refers to treatments that one intends to guide or direct by means of the prognostic model being developed or validated. For example, CVD prediction models are used to guide the prescription of lipid-lowering medication, as well as direct targeted advice about lifestyle changes to high-risk individuals. “Background treatments” refer to any other treatment that an individual receives during a prognostic study. This could, for example, include treatments that are part of routine medical care or changes an individual makes to their lifestyle. Figure 1 outlines the different stages where treatments may be used in a prognostic study.

#### Guided treatments

Prognostic models are often used to guide or direct the initiation of certain treatments or interventions. In this case, a prognostic model should estimate the risk of developing a certain outcome *if individuals were to remain untreated* with this particular treatment (so-called untreated risk prediction) [1, 8, 10]. If this particular, “guided” treatment is given to study participants after the predictors are measured but before the ascertainment of the outcome (henceforth, we refer to this as “treatment drop-in”, see Fig. 1), the chance of treated individuals developing the outcome of interest will be decreased. Crucially, the outcomes measured in the study will no longer represent the untreated outcomes that the model is designed to predict. It follows that models developed using data from individuals who received guided treatments will provide biased underestimates of (untreated) risks in future individuals, if treatment use is ignored [8]. In validation studies, models will incorrectly appear to overestimate risk if applied in individuals that receive the specific guided treatment [8, 11].

#### Background treatments

Participants in a prognostic study commonly receive risk-lowering treatments during follow-up as a part of routine care. As in the case of guided treatments, if these “background” treatments are effective in lowering the risk of the outcome under prediction, we can expect a reduction in the probability of treated individuals developing the outcome of interest. However, unlike with guided treatments, the outcomes measured in the study still reflect the outcome under prediction. Background treatments should instead be considered to be a part of the case-mix of participants in study. Provided the pattern of treatment use, and the effect of the treatment on the outcome risk, is consistent across populations, differences between model performance in the development cohort new populations should not be due to treatment use. However, background treatment use and effectiveness may vary between settings. For example, a model developed in a setting where everyone received some standard (effective) treatment during follow-up may not be transportable to a different population where that intervention is not available, or a less effective alternative treatment is routinely used. In this case, the predicted probabilities provided by the model in this new population will be too low.

#### Examples

We illustrate the distinction between different types of treatment with two hypothetical examples, from two different clinical domains.

##### Example 1:

A model is developed to predict six-month mortality risk in patients with end-stage renal disease (ERD) in the absence of a kidney transplantation. The model will be used to help decide which future patients will receive a kidney transplant. In the development cohort, all patients began risk-lowering haemodialysis after enrolment as a part of routine care, and a subset of patients additionally received a kidney transplant.

##### Example 2:

A validation study is conducted to evaluate an existing prognostic model for the prediction of five-year CVD risk in the general population. The model is used in practice to decide whether lipid-lowering drugs (statins) will be prescribed. Several individuals in the study were prescribed risk-lowering statins and were recommended to modify their lifestyle based on their predicted CVD risk. In addition, a number of patients took other risk-lowering medications (e.g. aspirin) as a part of routine care.

In both examples, some study participants initiated one or more treatments or interventions after predictor measurements were taken. In example 1, we can consider haemodialysis to be a “background” treatment, as described above, which requires no further consideration for model development. However, the model may need to be recalibrated for settings where haemodialysis is not a part of usual care or where a substantial proportion of patients receive some other type of (e.g. peritoneal) dialysis. In contrast, kidney transplant, a treatment guided by predictions made by the model, could bias model development. The outcomes measured in individuals who received a transplant during follow-up do not reflect our outcome of interest: six-month mortality without kidney transplantation. Not taking this into account in model development will lead to a prediction model that actually underestimates the risk of mortality without transplantation in future patients with ERD.

In example 2, the use of medications such as aspirin can be considered as background treatment that will not affect the validity of the validation study. It mayhowever explain model miscalibration in the validation cohort if the pattern of use or the effectiveness of these treatments is different from those of the development cohort. With regard to lipid-lowering medication, ideally one would validate the model in individuals who have not received lipid-lowering medication during follow-up. As high-risk individuals received statins in the study, their risk of a CVD event in the study is lower than it would have been, had they remained untreated. In this example, lifestyle changes merit separate attention. If the model is used in practice, as with statins, to help target lifestyle advice to high-risk individuals, this treatment should not be ignored in the validation study. However, many individuals may have modified their lifestyles independent of any targeted advice, in which case, lifestyle changes could be viewed as a background treatment.

To summarise, when treatments are initiated in participants after the moment of prognostication (see Fig. 1), the risk-lowering effects of these treatments may impact on model development or validation. We propose that the intended use and this kind of risk predictions a model aims to provide (i.e. prognosis with or without treatment), as well as the types of treatments (guided or background) used in a data set or study, are key factors that determine how treatments may impact on prognostic model development or validation. For further details on the challenges of treatment use and how to account for them in prognostic model development and validation, see [8] and [11], respectively, and further guidance can be found in Table 1 (see below).

**Table 1:** General characteristics of the included articles

Characteristics of included studies (*n* = 302)	
Study type*	
Development	124
Validation	146
Incremental value assessment	135
Over a set of core predictors	81
Design of study used for prognostic modelling	
Observational	286
Randomised trial	16
Follow-up period (years)	10, (6, 12); 15%^a^
Prediction horizon (years)	10, (8, 10); 12%^a^

*One article may have multiple study types (e.g. the development and validation of a model); thus values do not sum to the total number of included articles

^a^Values represent as follows: median (lower quartile, upper quartile), percentage of studies that did not report this information

### A review of treatment use in published prognostic model studies

To provide insight into the extent to which treatment use has been addressed in the development and validation of prognostic models, we used a previously conducted systematic review of the reporting and analysis of prognostic models for predicting the risk of the future occurrence of CVD outcomes in the general population [12]. A completed PRISMA checklist for this review is found in Additional file 1.

#### Data sources, search, and study selection

In brief, a search was performed on 1 June 2013 in MEDLINE and EMBASE to identify original research articles reporting the development (derivation of a new model) or external validation (evaluation of an existing model in a new population) of a prognostic model and “incremental value studies”, in which the additional value of a certain predictor or (bio)marker was assessed on top of either an existing risk score or a model consisting of a core set of conventional predictors (e.g. age, sex, smoking, systolic blood pressure, cholesterol, diabetes).

Titles and abstracts were first screened for eligibility, and subsequent full-text screening was conducted. Publications were considered for inclusion if they were original articles that reported cardiovascular risk prognostic modelling in a general population setting. Full details of the search strategy and in-/exclusion criteria can be found in the original review [12].

#### Data extraction

Directed by the CHARMS checklist [13], a list of key items (Additional file 2) for extraction was derived for the current review by one author (RP) and updated after group consideration (RP, LMP, RHHG, JAAGD, KGMM). As the aim of this review is to provide an overview of research practice and reporting, study quality and risk of bias assessment was not conducted. Independent data extraction was piloted among three authors (RP, JAAGD, RHHG). The remaining data extraction was conducted by one author (RP), and any queries were discussed primarily with one author (JAAGD), and then two other authors (LMP, RHHG) until a consensus was reached.

General study characteristics were extracted for each article, including the study design used to collect data, the start and end dates of participant data collection and the prediction horizons of reported models. Relevant treatments or interventions for cardiovascular disease prevention were defined prior to data extraction and broadly divided into three classes: pharmacological treatments (notably antihypertensive, lipid-lowering and antithrombotic medication), cardiovascular surgical interventions (e.g. coronary revascularization, carotid endarterectomy), and lifestyle interventions. While the term “lifestyle interventions” can refer to changes in a diverse range of modifiable risk factors, we defined this in our review as the reporting of active modifications to exercise, nutritional or smoking habits, as a part of a programme or following physician recommendations. All reported information on treatment use and how it was considered in the analysis was extracted (for full details, see Additional file 2).

## Results of the literature review

### General characteristics of included articles

**Fig. 2:**
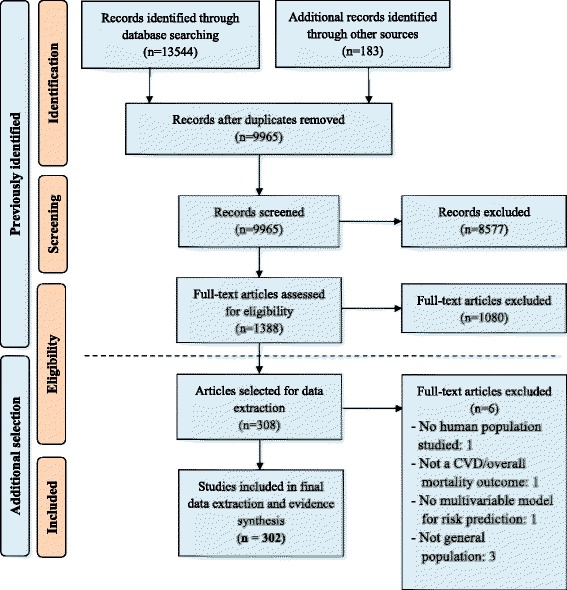
A flow diagram of article inclusion and exclusion

The search of the original systematic review identified 9965 unique records, of which 1388 were found to be relevant following title and abstract screening, as previously reported [12]. After full-text screening for eligibility, 302 articles were included for review (Additional file 3). A summary of the article inclusion process is presented in Fig. 2.

The final set of articles includes publications from 102 different journals. Publication dates ranged from 1967 to 2013 and 157 articles (52%) were published from 2009 onwards. Participant data collection ranged from as early as 1948 until 2011. Further details are presented in Table 2.

**Table 2:** Reporting of treatment use by study type

Reported treatment	Overall (*n* = 302) (%)^a^	Development studies (*n* = 124) (%)	Incremental value studies (*n* = 135) (%)	Validation studies (*n* = 146) (%)
Medication use (any)	135 (45)	45 (36)	73 (54)	62 (41)
Antihypertensive	122 (41)	40 (32)	66 (49)	58 (38)
Lipid-lowering	81 (27)	24 (19)	47 (33)	38 (26)
Antithrombotic/anticoagulant	17 (6)	2 (2)	15 (11)	7 (5)
Lifestyle interventions	2 (1)	1 (1)	0	1 (1)
Surgical interventions	39 (13)	9 (7)	26 (19)	15 (11)

^a^One article may have multiple study types (e.g. the development and validation of a model); thus values in individual columns do not sum to the overall number of included articles. Articles may have reported multiple treatments and thus percentages in each column should not necessarily sum to 100%

### Reporting and handling of treatment use

**Fig. 3:**
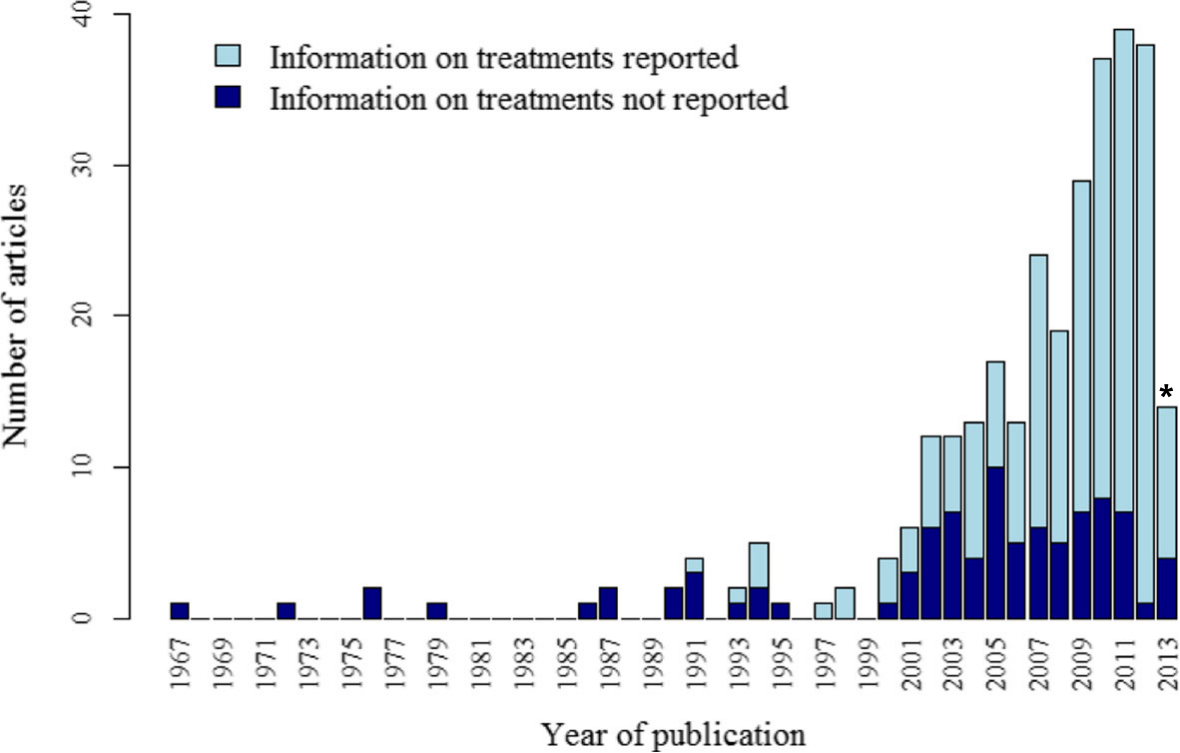
Reporting of treatment in CVD prognostic modelling studies over time. Articles were classified as having reported information on treatment if the use of at least one potentially risk-lowering treatment in the study was reported, or if the effect of a treatment on the study findings was discussed. (*) Articles were included up to June 2013; this column only represents treatment reporting during the first half of 2013

Overall, nearly one-third (91 articles, 30%) of the 302 included articles did not report any information about relevant preventative or therapeutic treatments. The reporting of treatments in prognostic modelling articles has increased over time, as illustrated in Fig. 3. Just over half of the articles published up until 2008 (81 articles, 56%) reported information about treatment, whereas from 2009 to June 2013, this increased (130 articles, 83%). Summaries of the reporting and handling of information about treatment use are presented in Tables 3 and 4, respectively.

**Table 3:** Handling of treatment in the analyses of prognostic model studies

Approach taken to account for treatment use	Development studies *n* = 124 (%)	Incremental value studies *n* = 135 (%)	Validation studies *n* = 146 (%)
Treated patients excluded from the analysis	20 (15)	53 (39)	38 (26)
Antihypertensive medication users	4 (3)	6 (4)	6 (4)
Lipid-lowering medication users	6 (5)	10 (7)	16 (11)
Other medication users	1 (1)	2 (1)	1 (1)
Lifestyle interventions	0	0	0
Patients who received surgery	14 (10)	39 (29)	22 (15)
Untreated patients-only sensitivity analysis	9 (7)	5 (4)	4 (3)
Stratification by treatment use	1 (1)	0	0
Treatment included in the outcome	23 (19)	58 (43)	35 (24)
Treatment modelled as a predictor	54 (44)	48 (59)^a^	–
Antihypertensive medication use	49 (40)	44 (54)^a^	–
Lipid-lowering medication use	12 (10)	15 (11)^a^	–
Other medication use	2 (2)	5 (4)^a^	–
Lifestyle interventions	1 (1)	0^a^	–
Surgical interventions	0	0^a^	–
Type of treatment information modelled			
Modelled directly (not a composite predictor^b^)	37 (30)	44 (54)^a^	–
Baseline treatment	41 (33)	36 (44)^a^	–
Changes in treatment during follow-up	0	0^a^	–
Treatment at the end of follow-up	0	1 (1)^a^	–
Not clearly reported	12 (10)	11 (8)	–
Statistical interactions with treatment considered	21 (17)	7 (5)^a^	–

^a^Only studies that assessed incremental value over a core set of individual predictors (*n* = 81) and thus had the opportunity to include treatment variables within the core set of predictors; studies that assessed incremental value over an existing prognostic model or risk score did not derive a new prediction model and are not included in the calculation

^b^Composite predictors are here defined as the combination of two or more variables (including treatment use) into a single predictor

**Table 4:** Addressing and reporting treatment use in prognostic model studies

Design	
Collect information on treatments used at the study baseline (see Fig. 1)	
Collect information on treatment drop-in or discontinuation during follow-up (see Fig. 1).	
If using readily available data (e.g. from an existing cohort or register), consider whether sufficient information on treatment use has been recorded.	
Analysis	
Model development	
Guided treatments: Consider explicitly including treatment use in the prognostic model. If a treatment was randomly allocated (e.g. data from an RCT), consider using only the subset of untreated individuals [8].	
Model validation	
Guided treatments: If treatments were randomly allocated, exclude treated individuals from the analysis. If treatment use is non-random (e.g. data from an observational study or register), consider first using inverse treatment probability weighting before validating the model in the untreated subset [11].	
Background treatments: Consider differences in treatment use between the development and validation cohorts when exploring the impact of case-mix on model performance [24–26].	
Reporting	
Report information on treatment use at baseline. List any treatments that may have affected the prognosis of individuals in the study and the absolute number (%) treated.	
Report information on effective treatments used during follow-up and, where relevant, the duration of treatment use.	
Discuss the potential impact of treatment use on the validity and transportability of the developed prognostic model or estimates of model performance.	

“Treatment” refers to any medical or non-medical intervention undertaken by an individual that lowers their risk of a certain outcome

#### Development studies

Of the 124 articles that reported the development of a new prognostic model, baseline information on treatment use was reported in 43 articles (35%). Six articles (5%) reported treatment use during follow-up, two (2%) reported changes in medication use during follow-up, four (3%) described incident surgical procedures (cardiovascular surgeries occurring after the study baseline) and in 11 articles (9%), the timing of treatments was unclear. Two articles reported that information on treatment was not available. Treatment use was most often accounted for in analyses by modelling treatment as a predictor (54 articles, 44%). Twenty articles (15%) excluded treated individuals from the analysis. Changes in treatment use during follow-up were not modelled.

#### Incremental value studies

In articles that reported the evaluation of the incremental value of a predictor over either a core set of predictors or an existing model, baseline information about treatment use was reported for 74 articles (55%). Changes in medication use were reported in three articles, and surgical procedures that occurred during follow-up were reported in 15 articles (11%). Five articles (4%) reported that information on treatment use was not available. Where incremental value was assessed over a set of core predictors, treatment use was accounted for most often by including treatment as one of the core predictors (48 articles, 59%). Fifty-three articles (39%) excluded treated individuals from analyses. Surgical outcomes were frequently modelled as a part of a composite endpoint (58 articles, 43%).

#### Validation studies

In studies that externally validated (evaluated) an existing CVD prognostic model, where reported, most information about treatment use was measured at baseline only (55 articles, 37%). No articles reported changes in medication use during follow-up. Four articles reported a lack of available data on treatment use. In addition, five articles (3%) presented information about treatment use in the population in which the model was originally developed, of which two reported differences of more than 10% in the proportion of baseline treatment users between the development study and the validation study. Another five articles (3%) commented on how differences between treatment use in the development and validation populations could have contributed to poor performance of the model upon validation. Medication use was accounted for exclusively by restricting analyses to untreated patients (38 articles, 26%). In addition, 35 articles (24%) accounted for incident surgical procedures by including surgery within the composite endpoint of their study.

## Discussion

### Findings from the literature review

The use of treatments in prognostic modelling studies has not been widely addressed in cardiovascular preventative medicine. While reporting has improved over the last decade, and the majority of cardiovascular prognostic modelling studies (211 articles, 70%) made at least one reference to treatment use, we found great heterogeneity in the kinds of information and level of detail that have been reported. Only 52% of studies that developed a model reported specific information about the use of risk-lowering treatments, similar to findings from a previous review in the field of cardiovascular medicine [6]. We also confirm that information beyond baseline antihypertensive medication use, information about other treatments, and changes in treatment use during follow-up are frequently not reported. In addition, we found the reporting or discussion of any differences between treatment use in validation studies and their respective development studies was poorer than that observed in an earlier review of external model validation studies, which found that 40% (31/78) of articles under study discussed differences in case-mix [14].

There are several possible explanations for the findings of the review. First, several articles used data collected during the pre-statin era [15], which may explain why the lipid-lowering medications were scarcely reported. However, effective medications such as aspirin and blood pressure-lowering medication have long been available, along with lifestyle interventions and some surgical procedures, which are also relevant to these studies. In addition, many articles reported a low prevalence of statin use at study baseline; in those situations, it may have been assumed that treatment would not have greatly influenced the predicted probabilities. However, treatment use can greatly change over time, as shown by one study validating the AHA/ACC Pooled Cohort Equations [16], which reported increases in antihypertensive medication use and statin use from 59.9 to 82.4% and 9.7 to 63.7%, respectively, over a 10-year follow-up period (1998–2007) [17]. Second, while only nine articles reported that data on treatments were not available in their studies, it might be that more studies were unable to obtain such data, especially follow-up information, as this may be more costly or difficult to collect. Finally, in some studies, treatments may not have been considered by the authors to be relevant to the prognostic question being addressed. One article did not model treatment effects on the grounds that “The prediction of initial CHD [coronary heart disease] events in a free-living population not on medication is emphasized” [18], i.e. the model was designed for use in individuals who are not already on treatment. However, as already discussed, this rationale does not take into account treatment drop-in that may have occurred during the follow-up period of the study.

The review is, to our knowledge, the first to give an overview of how treatment information has been reported and handled in prognostic model research. While other studies have broadly addressed related methodological issues [14], or have focussed on a single aspect of CVD modelling, such as model development [6], we provide comprehensive coverage of CVD prediction model studies and support this with a conceptual framework describing when and how treatments can affect a prognostic study. However, there are limitations within this study.

First, as the findings presented in the review are based on articles identified through a previously conducted systematic review, we are limited to providing information up to June 2013; more recent trends in cardiovascular prognostic modelling are not presented. Three important developments in the past 4 years include the ACC/AHA Pooled Cohort equations [16], the Globorisk CVD assessment tool [19], and the Qrisk-3 calculator [20], each developed as tools for the prediction of CVD in the general population. Among these three currently implemented CVD risk estimators, there is no clear consensus over how treatments should be taken into account in prognostic models for CVD; treatment use at baseline is modelled differently in each of the prognostic models, and none of the studies accounted for the effects of treatment drop-in. Thus, questions have been raised regarding the validity of these models and their respective validation studies [9, 21], and treatment use remains an issue at present. Furthermore, owing to the large number of included articles (> 100) published from 2009 onwards, our study provides a more up-to-date overview than previous findings [6]. As the CVD domain is a highly active field in prognostic model research, the presented results are likely optimistic for other clinical domains; we speculate that in other clinical domains, treatment use has received less attention. Second, this review focusses on a set of preventative and therapeutic treatments that modify cardiovascular risk, but may not describe all interventions that affect CVD risk. However, a detailed description is presented for the major classes of cardiovascular preventative treatments, particularly those recommended by medical guidelines. Third, as this is a review of reporting, we rely on what the authors decided to mention within the article and we cannot be entirely sure how treatment information has been collected in studies and the extent to which it has been considered by researchers. For example, limited information could be extracted about changes in lifestyle that may have affected prognostic modelling, as this was almost never explicitly reported.

### Suggestions for dealing with and reporting treatment use in prognostic model studies

Treatment use can potentially have a great impact on the reported accuracy of developed and validated prognostic models. Our review has identified that information about the use of treatments is often reported with insufficient detail to allow other researchers to evaluate the effect it may have had on the reported study findings, notably the expected predictive accuracy model in future populations. The TRIPOD statement [22, 23] has already made recommendations for the reporting of information on treatment use in prognostic model studies (Item 5c), but these can be strengthened on this aspect. We provide additional recommendations for the design, analysis, and reporting of prognostic model studies, to help improve the way that treatment use, in particular during follow-up, is addressed (Table 4).

Starting with the design of future prognostic studies, we suggest that information should be collected on both treatment use at the study baseline and during follow-up, to record any changes in treatment use over time that may have impacted on the prognosis of study participants. Existing databases should contain information with enough detail to allow researchers to account for treatment use in their analyses, where necessary (see “What do we mean by “treatment” and when is it a problem?” section). We provide initial recommendations on how different kinds of treatments can be taken into account when developing or validating a prediction model. This advice is based on a limited number of simulation studies, and in the absence of further simulations and empirical evidence, researchers must judge which approach will be most valid for their research. We do not provide specific guidance on how to account for complex changes in treatment use in a prognostic study, as more research is needed into the suitability of existing statistical methods. Finally, Table 1 provides, in accordance with the TRIPOD guidelines [23], recommendations for the minimum amount of detail that should be presented in reports of prognostic model studies. We encourage researchers to discuss the potential impact that treatment use in their study could have had on their results, including the expected accuracy of newly developed models.

## Conclusion

In conclusion, treatment use, if ignored, can raise concerns for the transportability and validity of prognostic models. Our review shows that while the importance of treatments for prognostic prediction has been recognised in many studies, reporting rarely covers all relevant treatments, and changes in treatment have hardly been acknowledged. Furthermore, we found no clear consensus within the published literature over how treatments should be considered in the analyses of prognostic studies. Efforts should be made to collect and report detailed information about treatment use, to allow future researchers and end users of prognostic models to more clearly identify any potential issues that treatment use may have introduced and to understand how a model should be validated and used in practice.

## Additional files

Additional file 1: PRISMA statement checklist. (DOC 62 kb)

Additional file 2: List of items for data extraction. (DOCX 17 kb)

Additional file 3: List of articles included in the literature review. (DOCX 58 kb)

Additional file 4: Table of extracted information from the review. (CSV 209 kb)

## Abbreviations

CHD: Coronary heart disease
CVD: Cardiovascular disease

## References

[1] Hemingway H, Croft P, Perel P, Hayden JA, Abrams K, Timmis A (2013). Prognosis research strategy (PROGRESS) 1: a framework for researching clinical outcomes. BMJ.

[2] Steyerberg EW, Moons KG, van der Windt DA, Hayden JA, Perel P, Schroter S (2013). Prognosis Research Strategy (PROGRESS) 3: prognostic model research. PLoS Med.

[3] Stone NJ, Robinson JG, Lichtenstein AH, Bairey Merz CN, Blum CB, Eckel RH (2014). 2013 ACC/AHA guideline on the treatment of blood cholesterol to reduce atherosclerotic cardiovascular risk in adults: a report of the American College of Cardiology/American Heart Association Task Force on Practice Guidelines. Circulation.

[4] National Institute for Health and Care Excellence. Lipid modification: cardiovascular risk assessment and the modification of blood lipids for the primary and secondary prevention of cardiovascular disease. NICE Clinical Guideline 181. London. 2014. 25340243

[5] Perk J, De Backer G, Gohlke H, Graham I, Reiner Z, Verschuren M (2012). European Guidelines on cardiovascular disease prevention in clinical practice (version 2012). The Fifth Joint Task Force of the European Society of Cardiology and Other Societies on cardiovascular disease prevention in clinical practice (constituted by representatives of nine societies and by invited experts). Eur Heart J.

[6] Liew SM, Doust J, Glasziou P (2011). Cardiovascular risk scores do not account for the effect of treatment: a review. Heart.

[7] Liew SM, Glasziou P. Risk prediction continue to ignore treatment effects. Br Med J Rapid Responses; 2010;340:c2442. http://www.bmj.com/rapid-response/2011/11/02/risk-prediction-continue-ignore-treatment-effects.

[8] Groenwold RH, Moons KG, Pajouheshnia R, Altman DG, Collins GS, Debray TP, et al. Explicit inclusion of treatment in prognostic modelling was recommended in observational and randomised settings. J Clin Epidemiol. 2016;78:90–100. 10.1016/j.jclinepi.2016.03.017 27045189

[9] Peek N, Sperrin M, Mamas M, Van Staa T, Buchan I. Hari Seldon, QRISK3, and the prediction paradox. BMJ. 2017;357:j2099. http://www.bmj.com/content/357/bmj.j2099/rr-0.

[10] Grobbee DE, Hoes AW. Clinical epidemiology. 2nd ed: Jones & Bartlett Publishers; London 2014.

[11] Pajouheshnia R, Peelen LM, Moons KGM, Reitsma JB, Groenwold RHH (2017). Accounting for treatment use when validating a prognostic model: a simulation study. BMC Med Res Methodol.

[12] Damen JA, Hooft L, Schuit E, Debray TP, Collins GS, Tzoulaki I (2016). Prediction models for cardiovascular disease risk in the general population: systematic review. BMJ.

[13] Moons KG, de Groot JA, Bouwmeester W, Vergouwe Y, Mallett S, Altman DG (2014). Critical appraisal and data extraction for systematic reviews of prediction modelling studies: the CHARMS checklist. PLoS Med.

[14] Collins GS, de Groot JA, Dutton S, Omar O, Shanyinde M, Tajar A (2014). External validation of multivariable prediction models: a systematic review of methodological conduct and reporting. BMC Med Res Methodol.

[15] Tobert JA (2003). Lovastatin and beyond: the history of the HMG-CoA reductase inhibitors. Nat Rev Drug Discov.

[16] Goff DC, Lloyd-Jones DM, Bennett G, Coady S, D'Agostino RB, Gibbons R (2014). 2013 ACC/AHA guideline on the assessment of cardiovascular risk: a report of the American College of Cardiology/American Heart Association Task Force on Practice Guidelines. Circulation.

[17] Chia YC, Lim HM, Ching SM (2014). Validation of the pooled cohort risk score in an Asian population—a retrospective cohort study. BMC Cardiovasc Disord.

[18] Wilson PW, D'Agostino RB, Levy D, Belanger AM, Silbershatz H, Kannel WB (1998). Prediction of coronary heart disease using risk factor categories. Circulation.

[19] Hajifathalian K, Ueda P, Lu Y, Woodward M, Ahmadvand A, Aguilar-Salinas CA (2015). A novel risk score to predict cardiovascular disease risk in national populations (Globorisk): a pooled analysis of prospective cohorts and health examination surveys. Lancet Diabetes Endocrinol.

[20] Hippisley-Cox J, Coupland C, Brindle P. Development and validation of QRISK3 risk prediction algorithms to estimate future risk of cardiovascular disease: prospective cohort study. BMJ. 2017;357 10.1136/bmj.j2099 PMC5441081 28536104

[21] Muntner P, Safford MM, Cushman M, Howard G (2014). Comment on the reports of over-estimation of ASCVD risk using the 2013 AHA/ACC risk equation. Circulation.

[22] Collins GS, Reitsma JB, Altman DG, Moons KG (2015). Transparent reporting of a multivariable prediction model for individual prognosis or diagnosis (TRIPOD): the TRIPOD statement. BMJ.

[23] Moons KG, Altman DG, Reitsma JB, Ioannidis JP, Macaskill P, Steyerberg EW (2015). Transparent reporting of a multivariable prediction model for individual prognosis or diagnosis (TRIPOD): explanation and elaboration. Ann Intern Med.

[24] Debray TP, Vergouwe Y, Koffijberg H, Nieboer D, Steyerberg EW, Moons KG (2015). A new framework to enhance the interpretation of external validation studies of clinical prediction models. J Clin Epidemiol.

[25] Riley RD, Ensor J, Snell KIE, Debray TPA, Altman DG, Moons KGM, et al. External validation of clinical prediction models using big datasets from e-health records or IPD meta-analysis: opportunities and challenges. BMJ. 2016;353 10.1136/bmj.i3140 PMC4916924 27334381

[26] Vergouwe Y, Moons KG, Steyerberg EW (2010). External validity of risk models: use of benchmark values to disentangle a case-mix effect from incorrect coefficients. Am J Epidemiol.

