# Supplementary material for: Treatment use in prognostic model research: a systematic review of cardiovascular prognostic studies
Source: Diagn Progn Res. 2017 Sep 26;1:15. doi: 10.1186/s41512-017-0015-0 (PMC6460846; doi:10.1186/s41512-017-0015-0)
Supplement: Supplementary file 2 — List of items for data extraction. (DOCX 17 kb) [file 41512_2017_15_MOESM2_ESM.docx]

Appendix table 1: List of items for data extraction

| 1. General study information |
| --- |
| General study aims. |
| Study type. |
| For incremental value (IV) studies:  Is IV assessed over an existing model or a new model containing conventional predictors? |
| Study design. |
| Start of data collection. |
| End of data collection. |
| Length of follow-up. |
| Intended prediction horizon. |
| 2. Reporting of treatment-specific information |
| Where in the article is information about treatment reported? |
| Is a treatment included within the definition of the outcome?   - If so, give details. |
| Is a treatment included within the definition of a predictor variable (composite predictor)?   - If so, give details. |
| Is use of any of the following treatments reported (E.g. proportion of users)?   - Cholesterol/lipid-lowering medication. - Blood pressure-lowering/antihypertensive medication. - Antithrombotic/anticoagulant medication. - Lifestyle modification advice/programmes. - Cardiovascular procedure/surgery. |
| If no specific details about treatment use are reported, is the collection of information about treatment use clearly reported (I.e. in the methods)? |
| At which stage of data collection was reported information measured (E.g. at baseline or during follow-up)? |
| If follow-up information is reported,   - Are incident surgical procedures reported? - Are changes in medication use during follow-up reported? |
| Is treatment explicitly mentioned as part of the participant eligibility criteria?   - If so, which treatments? |
| Is the relevance of treatment explicitly discussed (with reference to the performance or generalizability of the model)?   - If so, provide details. |
| For validation studies:  Is treatment uses explicitly reported for both validation study population and the original development study population?   - If so,   - Is there a difference in treatment use between the two sets (difference in proportion treated greater than 10%)?   - Are the implications of any differences discussed?     - If so, give details. |
| 3. Accounting for treatment use in the analysis |
| If treatments are not accounted for in the analysis, is a reason given for why this is so?   - If so, give details. |
| Is the analysis restricted according to use of a treatment (I.e. Are treated individuals excluded?)?   - If so,   - Restricted on which treatment?   - Is restriction based on baseline status or treatment during follow-up?   - Is this a part of a sensitivity analysis? |
| Is treatment modelled as a predictor?   - If so,   - Which treatments are modelled?   - Give details on the exact definition.   - Is treatment modelled within a composite predictor?   - Which kind of treatment information is modelled: baseline, follow-up, both?   - Is treatment modelled using more advanced statistical techniques (E.g. as a time-varying covariate)?     - If so, give details.   - Are treatment interactions with other variables modelled?   - Is treatment included as a predictor in the final model?     - If not, what is the rationale behind not including the modelled treatment in the final model?   - Is a treatment modelled alongside any associated condition (I.e. blood pressure-lowering medication and blood pressure)? |
| Are analyses stratified according to treatment use? |
| For validation studies:  Is the existing model recalibrated/updated with the specific aim of accounting for treatment use? |
