# Supplementary material for: Treatment use in prognostic model research: a systematic review of cardiovascular prognostic studies
Source: Diagn Progn Res. 2017 Sep 26;1:15. doi: 10.1186/s41512-017-0015-0 (PMC6460846; doi:10.1186/s41512-017-0015-0)
Supplement: Supplementary file 3 — List of articles included in the literature review. (DOCX 58 kb) [file 41512_2017_15_MOESM3_ESM.docx]

Appendix table 2: List of articles included in the review

| 1. | Third Report of the National Cholesterol Education Program (NCEP) Expert Panel on Detection, Evaluation, and Treatment of High Blood Cholesterol in Adults (Adult Treatment Panel III) final report. Circulation. 2002;106(25):3143-421. Epub 2002/12/18. |
| --- | --- |
| 2. | Aktas MK, Ozduran V, Pothier CE, Lang R, Lauer MS. Global risk scores and exercise testing for predicting all-cause mortality in a preventive medicine program. JAMA. 2004;292(12):1462-8. |
| 3. | Alssema M, Newson RS, Bakker SJL, Stehouwer CDA, Heymans MW, Nijpels G, et al. One risk assessment tool for cardiovascular disease, type 2 diabetes, and chronic kidney disease. Diabetes Care. 2012;35(4):741-8. |
| 4. | Anderson KM, Odell PM, Wilson PW, Kannel WB. Cardiovascular disease risk profiles. Am Heart J. 1991;121(1 Pt 2):293-8. Epub 1991/01/01. |
| 5. | Anderson KM, Wilson PW, Odell PM, Kannel WB. An updated coronary risk profile. A statement for health professionals. Circulation. 1991;83(1):356-62. Epub 1991/01/01. |
| 6. | Araujo AB, Hall SA, Ganz P, Chiu GR, Rosen RC, Kupelian V, et al. Does erectile dysfunction contribute to cardiovascular disease risk prediction beyond the Framingham risk score? J Am Coll Cardiol. 2010;55(4):350-6. |
| 7. | Arima H, Yonemoto K, Doi Y, Ninomiya T, Hata J, Tanizaki Y, et al. Development and validation of a cardiovascular risk prediction model for Japanese: the Hisayama study. Hypertens Res. 2009;32(12):1119-22. |
| 8. | Asayama K, Ohkubo T, Sato A, Hara A, Obara T, Yasui D, et al. Proposal of a risk-stratification system for the Japanese population based on blood pressure levels: the Ohasama study. Hypertens Res. 2008;31(7):1315-22. Epub 2008/10/30. |
| 9. | Asia Pacific Cohort Studies Collaboration. Coronary risk prediction for those with and without diabetes. Eur J Cardiovasc Prev Rehabil. 2006;13(1):30-6. Epub 2006/02/02. |
| 10. | Asia Pacific Cohort Studies Collaboration, Barzi F, Patel A, Gu D, Sritara P, Lam TH, et al. Cardiovascular risk prediction tools for populations in Asia. J Epidemiol Community Health. 2007;61(2):115-21. |
| 11. | Aslibekyan S, Campos H, Loucks EB, Linkletter CD, Ordovas JM, Baylin A. Development of a cardiovascular risk score for use in low- and middle-income countries. J Nutr. 2011;141(7):1375-80. |
| 12. | Asselbergs FW, Hillege HL, van Gilst WH. Framingham score and microalbuminuria: combined future targets for primary prevention? Kidney Int Suppl. 2004(92):S111-4. |
| 13. | Assmann G, Cullen P, Schulte H. Simple scoring scheme for calculating the risk of acute coronary events based on the 10-year follow-up of the prospective cardiovascular Munster (PROCAM) study. Circulation. 2002;105(3):310-5. Epub 2002/01/24. |
| 14. | Assmann G, Schulte H, Cullen P, Seedorf U. Assessing risk of myocardial infarction and stroke: new data from the Prospective Cardiovascular Munster (PROCAM) study. Eur J Clin Invest. 2007;37(12):925-32. |
| 15. | Assmann G, Schulte H, Seedorf U. Cardiovascular risk assessment in the metabolic syndrome: results from the Prospective Cardiovascular Munster (PROCAM) Study. Int J Obes. 2008;32 Suppl 2:S11-6. |
| 16. | Badheka AO, Patel N, Tuliani TA, Rathod A, Marzouka GR, Zalawadiya S, et al. Electrocardiographic abnormalities and reclassification of cardiovascular risk: insights from NHANES-III. Am J Med. 2013;126(4):319-26.e2. |
| 17. | Baik I, Cho NH, Kim SH, Shin C. Dietary information improves cardiovascular disease risk prediction models. Eur J Clin Nutr. 2013;67(1):25-30. |
| 18. | Baldassarre D, Hamsten A, Veglia F, de Faire U, Humphries SE, Smit AJ, et al. Measurements of carotid intima-media thickness and of interadventitia common carotid diameter improve prediction of cardiovascular events: results of the IMPROVE (Carotid Intima Media Thickness [IMT] and IMT-Progression as Predictors of Vascular Events in a High Risk European Population) study. J Am Coll Cardiol. 2012;60(16):1489-99. |
| 19. | Balkau B, Hu G, Qiao Q, Tuomilehto J, Borch-Johnsen K, Pyorala K, et al. Prediction of the risk of cardiovascular mortality using a score that includes glucose as a risk factor. The DECODE Study. Diabetologia. 2004;47(12):2118-28. |
| 20. | Bare LA, Morrison AC, Rowland CM, Shiffman D, Luke MM, Iakoubova OA, et al. Five common gene variants identify elevated genetic risk for coronary heart disease. Genet Med. 2007;9(10):682-9. |
| 21. | Barroso LC, Muro EC, Herrera ND, Ochoa GF, Hueros JIC, Buitrago F. Performance of the Framingham and SCORE cardiovascular risk prediction functions in a non-diabetic population of a Spanish health care centre: a validation study. Scand J Prim Health Care. 2010;28(4):242-8. |
| 22. | Bastuji-Garin S, Deverly A, Moyse D, Castaigne A, Mancia G, de Leeuw PW, et al. The Framingham prediction rule is not valid in a European population of treated hypertensive patients. J Hypertens. 2002;20(10):1973-80. |
| 23. | Baxi NS, Jackson JL, Ritter J, Sessums LL. How well do the Framingham risk factors correlate with diagnoses of ischemic heart disease and cerebrovascular disease in a military beneficiary cohort? Mil Med. 2011;176(4):408-13. |
| 24. | Becker CR, Majeed A, Crispin A, Knez A, Schoepf UJ, Boekstegers P, et al. CT measurement of coronary calcium mass: impact on global cardiac risk assessment. Eur Radiol. 2005;15(1):96-101. |
| 25. | Beer C, Alfonso H, Flicker L, Norman PE, Hankey GJ, Almeida OP. Traditional risk factors for incident cardiovascular events have limited importance in later life compared with the health in men study cardiovascular risk score. Stroke. 2011;42(4):952-9. |
| 26. | Bell K, Hayen A, McGeechan K, Neal B, Irwig L. Effects of additional blood pressure and lipid measurements on the prediction of cardiovascular risk. Eur J Prev Cardiol. 2012;19(6):1474-85. |
| 27. | Berard E, Bongard V, Arveiler D, Amouyel P, Wagner A, Dallongeville J, et al. Ten-year risk of all-cause mortality: assessment of a risk prediction algorithm in a French general population. Eur J Epidemiol. 2011;26(5):359-68. |
| 28. | Berry JD, Lloyd-Jones DM, Garside DB, Greenland P. Framingham risk score and prediction of coronary heart disease death in young men. Am Heart J. 2007;154(1):80-6. |
| 29. | Bhopal R, Fischbacher C, Vartiainen E, Unwin N, White M, Alberti G. Predicted and observed cardiovascular disease in South Asians: application of FINRISK, Framingham and SCORE models to Newcastle Heart Project data. J Public Health. 2005;27(1):93-100. |
| 30. | Bineau S, Dufouil C, Helmer C, Ritchie K, Empana J-P, Ducimetiere P, et al. Framingham stroke risk function in a large population-based cohort of elderly people: the 3C study. Stroke. 2009;40(5):1564-70. |
| 31. | Boland B, De Muylder R, Goderis G, Degryse J, Gueuning Y, Paulus D, et al. Cardiovascular prevention in general practice: development and validation of an algorithm. Acta Cardiol. 2004;59(6):598-605. |
| 32. | Bolton JL, Stewart MCW, Wilson JF, Anderson N, Price JF. Improvement in Prediction of Coronary Heart Disease Risk over Conventional Risk Factors Using SNPs Identified in Genome-Wide Association Studies. PLoS ONE. 2013;8(2). |
| 33. | Boudik F, Reissigova J, Hrach K, Tomeckova M, Bultas J, Anger Z, et al. Primary prevention of coronary artery disease among middle aged men in Prague: twenty-year follow-up results. Atherosclerosis. 2006;184(1):86-93. Epub 2005/11/19. |
| 34. | Boyar A. Creating a web application that combines Framingham risk with Electron Beam CT Coronary Calcium Score to calculate a new event risk. J Thorac Imaging. 2006;21(1):91-6. |
| 35. | Bozorgmanesh M, Hadaegh F, Azizi F. Predictive accuracy of the 'Framingham's general CVD algorithm' in a Middle Eastern population: Tehran Lipid and Glucose Study. Int J Clin Pract. 2011;65(3):264-73. |
| 36. | Brand RJ, Rosenman RH, Sholtz RI, Friedman M. Multivariate prediction of coronary heart disease in the Western Collaborative Group Study compared to the findings of the Framingham study. Circulation. 1976;53(2):348-55. Epub 1976/02/01. |
| 37. | Braun J, Bopp M, Faeh D. Blood glucose may be an alternative to cholesterol in CVD risk prediction charts. Cardiovasc Diabetol. 2013;12(1). |
| 38. | Brautbar A, Ballantyne CM, Lawson K, Nambi V, Chambless L, Folsom AR, et al. Impact of adding a single allele in the 9p21 locus to traditional risk factors on reclassification of coronary heart disease risk and implications for lipid-modifying therapy in the Atherosclerosis Risk in Communities study. Circ Cardiovasc Genet. 2009;2(3):279-85. |
| 39. | Brautbar A, Pompeii LA, Dehghan A, Ngwa JS, Nambi V, Virani SS, et al. A genetic risk score based on direct associations with coronary heart disease improves coronary heart disease risk prediction in the Atherosclerosis Risk in Communities (ARIC), but not in the Rotterdam and Framingham Offspring, Studies. Atherosclerosis. 2012;223(2):421-6. |
| 40. | Brindle P, Emberson J, Lampe F, Walker M, Whincup P, Fahey T, et al. Predictive accuracy of the Framingham coronary risk score in British men: prospective cohort study. BMJ. 2003;327(7426):1267. Epub 2003/12/04. |
| 41. | Brindle P, May M, Gill P, Cappuccio F, D'Agostino R, Sr., Fischbacher C, et al. Primary prevention of cardiovascular disease: a web-based risk score for seven British black and minority ethnic groups. Heart. 2006;92(11):1595-602. Epub 2006/06/10. |
| 42. | Brindle PM, McConnachie A, Upton MN, Hart CL, Davey Smith G, Watt GCM. The accuracy of the Framingham risk-score in different socioeconomic groups: a prospective study. Br J Gen Pract. 2005;55(520):838-45. |
| 43. | Brunner EJ, Shipley MJ, Marmot MG, Kivimaki M, Witte DR. Do the Joint British Society (JBS2) guidelines on prevention of cardiovascular disease with respect to plasma glucose improve risk stratification in the general population? Prospective cohort study. Diabet Med. 2010;27(5):550-5. |
| 44. | Buitrago F, Calvo-Hueros JI, Canon-Barroso L, Pozuelos-Estrada G, Molina-Martinez L, Espigares-Arroyo M, et al. Original and REGICOR Framingham functions in a nondiabetic population of a Spanish health care center: a validation study. Ann Fam Med. 2011;9(5):431-8. |
| 45. | Canoui-Poitrine F, Luc G, Mallat Z, Machez E, Bingham A, Ferrieres J, et al. Systemic chemokine levels, coronary heart disease, and ischemic stroke events: the PRIME study. Neurology. 2011;77(12):1165-73. |
| 46. | Cao JJ, Arnold AM, Manolio TA, Polak JF, Psaty BM, Hirsch CH, et al. Association of carotid artery intima-media thickness, plaques, and C-reactive protein with future cardiovascular disease and all-cause mortality: The cardiovascular health study. Circulation. 2007;116(1):32-8. |
| 47. | Chamberlain AM, Agarwal SK, Folsom AR, Soliman EZ, Chambless LE, Crow R, et al. A clinical risk score for atrial fibrillation in a biracial prospective cohort (from the Atherosclerosis Risk in Communities [ARIC] study). Am J Cardiol. 2011;107(1):85-91. |
| 48. | Chambless LE, Folsom AR, Sharrett AR, Sorlie P, Couper D, Szklo M, et al. Coronary heart disease risk prediction in the Atherosclerosis Risk in Communities (ARIC) study. J Clin Epidemiol. 2003;56(9):880-90. Epub 2003/09/25. |
| 49. | Chambless LE, Heiss G, Shahar E, Earp MJ, Toole J. Prediction of ischemic stroke risk in the Atherosclerosis Risk in Communities Study.[Erratum appears in Am J Epidemiol. 2004 Nov 1;160(9):927]. Am J Epidemiol. 2004;160(3):259-69. |
| 50. | Chamnan P, Simmons RK, Hori H, Sharp S, Khaw K-T, Wareham NJ, et al. A simple risk score using routine data for predicting cardiovascular disease in primary care. Br J Gen Pract. 2010;60(577):e327-34. |
| 51. | Chen L, Tonkin AM, Moon L, Mitchell P, Dobson A, Giles G, et al. Recalibration and validation of the SCORE risk chart in the Australian population: the AusSCORE chart. Eur J Cardiovasc Prev Rehabil. 2009;16(5):562-70. |
| 52. | Chien KL, Hsu HC, Su TC, Chang WT, Chen PC, Sung FC, et al. Constructing a point-based prediction model for the risk of coronary artery disease in a Chinese community: A report from a cohort study in Taiwan. Int J Cardiol. 2012;157(2):263-8. |
| 53. | Chien KL, Su TC, Hsu HC, Chang WT, Chen PC, Sung FC, et al. Constructing the prediction model for the risk of stroke in a Chinese population: report from a cohort study in Taiwan. Stroke. 2010;41(9):1858-64. Epub 2010/07/31. |
| 54. | Chironi G, Simon A, Megnien J-L, Sirieix M-E, Mousseaux E, Pessana F, et al. Impact of coronary artery calcium on cardiovascular risk categorization and lipid-lowering drug eligibility in asymptomatic hypercholesterolemic men. Int J Cardiol. 2011;151(2):200-4. |
| 55. | Church TS, Levine BD, McGuire DK, Lamonte MJ, Fitzgerald SJ, Cheng YJ, et al. Coronary artery calcium score, risk factors, and incident coronary heart disease events. Atherosclerosis. 2007;190(1):224-31. |
| 56. | Ciampi A, Courteau J, Niyonsenga T, Xhignesse M, Lussier-Cacan S, Roy M. Family history and the risk of coronary heart disease: comparing predictive models. Eur J Epidemiol. 2001;17(7):609-20. Epub 2002/06/28. |
| 57. | Collins GS, Altman DG. An independent external validation and evaluation of QRISK cardiovascular risk prediction: a prospective open cohort study. BMJ. 2009;339:b2584. |
| 58. | Collins GS, Altman DG. An independent and external validation of QRISK2 cardiovascular disease risk score: a prospective open cohort study. BMJ. 2010;340:c2442. |
| 59. | Collins GS, Altman DG. Predicting the 10 year risk of cardiovascular disease in the United Kingdom: independent and external validation of an updated version of QRISK2. BMJ. 2012;344:e4181. Epub 2012/06/23. |
| 60. | Comin E, Solanas P, Cabezas C, Subirana I, Ramos R, Gene-Badia J, et al. Estimating cardiovascular risk in Spain using different algorithms. Rev Esp Cardiol. 2007;60(7):693-702. |
| 61. | Conroy RM, Pyorala K, Fitzgerald AP, Sans S, Menotti A, De Backer G, et al. Estimation of ten-year risk of fatal cardiovascular disease in Europe: the SCORE project. Eur Heart J. 2003;24(11):987-1003. Epub 2003/06/06. |
| 62. | Cook NR, Buring JE, Ridker PM. The effect of including C-reactive protein in cardiovascular risk prediction models for women. Ann Intern Med. 2006;145(1):21-9. |
| 63. | Cook NR, Paynter NP, Eaton CB, Manson JE, Martin LW, Robinson JG, et al. Comparison of the Framingham and Reynolds Risk scores for global cardiovascular risk prediction in the multiethnic Women's Health Initiative. Circulation. 2012;125(14):1748-56, S1-11. |
| 64. | Cooney MT, Dudina A, De Bacquer D, Fitzgerald A, Conroy R, Sans S, et al. How much does HDL cholesterol add to risk estimation? A report from the SCORE Investigators. Eur J Cardiovasc Prev Rehabil. 2009;16(3):304-14. |
| 65. | Cooney MT, Vartiainen E, Laatikainen T, Joulevi A, Dudina A, Graham I. Simplifying cardiovascular risk estimation using resting heart rate. Eur Heart J. 2010;31(17):2141-7. |
| 66. | Cooper JA, Miller GJ, Humphries SE. A comparison of the PROCAM and Framingham point-scoring systems for estimation of individual risk of coronary heart disease in the Second Northwick Park Heart Study. Atherosclerosis. 2005;181(1):93-100. |
| 67. | Cournot M, Bura A, Cambou J-P, Taraszkiewicz D, Maloizel J, Galinier M, et al. Arterial ultrasound screening as a tool for coronary risk assessment in asymptomatic men and women. Angiology. 2012;63(4):282-8. |
| 68. | Cournot M, Taraszkiewicz D, Cambou J-P, Galinier M, Boccalon H, Hanaire-Broutin H, et al. Additional prognostic value of physical examination, exercise testing, and arterial ultrasonography for coronary risk assessment in primary prevention. Am Heart J. 2009;158(5):845-51. |
| 69. | Cournot M, Taraszkiewicz D, Galinier M, Chamontin B, Boccalon H, Hanaire-Broutin H, et al. Is exercise testing useful to improve the prediction of coronary events in asymptomatic subjects? Eur J Cardiovasc Prev Rehabil. 2006;13(1):37-44. |
| 70. | Cross DS, McCarty CA, Hytopoulos E, Beggs M, Nolan N, Harrington DS, et al. Coronary risk assessment among intermediate risk patients using a clinical and biomarker based algorithm developed and validated in two population cohorts. Curr Med Res Opin. 2012;28(11):1819-30. |
| 71. | Cushman M, Arnold AM, Psaty BM, Manolio TA, Kuller LH, Burke GL, et al. C-reactive protein and the 10-year incidence of coronary heart disease in older men and women: the cardiovascular health study. Circulation. 2005;112(1):25-31. |
| 72. | D'Agostino RB, Sr., Grundy S, Sullivan LM, Wilson P. Validation of the Framingham coronary heart disease prediction scores: results of a multiple ethnic groups investigation. JAMA. 2001;286(2):180-7. Epub 2001/07/13. |
| 73. | D'Agostino RB, Russell MW, Huse DM, Ellison RC, Silbershatz H, Wilson PW, et al. Primary and subsequent coronary risk appraisal: new results from the Framingham study. Am Heart J. 2000;139(2 Pt 1):272-81. Epub 2000/01/29. |
| 74. | D'Agostino RB, Sr., Vasan RS, Pencina MJ, Wolf PA, Cobain M, Massaro JM, et al. General cardiovascular risk profile for use in primary care: the Framingham Heart Study. Circulation. 2008;117(6):743-53. Epub 2008/01/24. |
| 75. | D'Agostino RB, Wolf PA, Belanger AJ, Kannel WB. Stroke risk profile: adjustment for antihypertensive medication. The Framingham Study. Stroke. 1994;25(1):40-3. Epub 1994/01/01. |
| 76. | Davies RW, Dandona S, Stewart AFR, Chen L, Ellis SG, Tang WHW, et al. Improved prediction of cardiovascular disease based on a panel of single nucleotide polymorphisms identified through genome-wide association studies. Circ Cardiovasc Genet. 2010;3(5):468-74. |
| 77. | De Bacquer D, De Backer G. Predictive ability of the SCORE Belgium risk chart for cardiovascular mortality. Int J Cardiol. 2010;143(3):385-90. |
| 78. | de la Iglesia B, Potter JF, Poulter NR, Robins MM, Skinner J. Performance of the ASSIGN cardiovascular disease risk score on a UK cohort of patients from general practice. Heart. 2011;97(6):491-9. |
| 79. | de Ruijter W, Westendorp RGJ, Assendelft WJJ, den Elzen WPJ, de Craen AJM, le Cessie S, et al. Use of Framingham risk score and new biomarkers to predict cardiovascular mortality in older people: population based observational cohort study. BMJ. 2009;338:a3083. |
| 80. | DECODE Study Group. Does diagnosis of the metabolic syndrome detect further men at high risk of cardiovascular death beyond those identified by a conventional cardiovascular risk score? The DECODE Study. Eur J Cardiovasc Prev Rehabil. 2007;14(2):192-9. Epub 2007/04/21. |
| 81. | Denes P, Larson JC, Lloyd-Jones DM, Prineas RJ, Greenland P. Major and minor ECG abnormalities in asymptomatic women and risk of cardiovascular events and mortality. JAMA. 2007;297(9):978-85. |
| 82. | Detrano R, Guerci AD, Carr JJ, Bild DE, Burke G, Folsom AR, et al. Coronary calcium as a predictor of coronary events in four racial or ethnic groups. N Engl J Med. 2008;358(13):1336-45. |
| 83. | Dhamoon MS, Moon YP, Paik MC, Sacco RL, Elkind MSV. The inclusion of stroke in risk stratification for primary prevention of vascular events: the Northern Manhattan Study. Stroke. 2011;42(10):2878-82. |
| 84. | Ding K, Bailey KR, Kullo IJ. Genotype-informed estimation of risk of coronary heart disease based on genome-wide association data linked to the electronic medical record. BMC Cardiovasc Disord. 2011;11:66. |
| 85. | Diverse Populations Collaborative Group. Prediction of mortality from coronary heart disease among diverse populations: is there a common predictive function? Heart. 2002;88(3):222-8. Epub 2002/08/16. |
| 86. | Donfrancesco C, Palmieri L, Cooney M-T, Vanuzzo D, Panico S, Cesana G, et al. Italian cardiovascular mortality charts of the CUORE project: are they comparable with the SCORE charts? Eur J Cardiovasc Prev Rehabil. 2010;17(4):403-9. |
| 87. | Drawz PE, Baraniuk S, Davis BR, Brown CD, Colon PJ, Sr., Cujyet AB, et al. Cardiovascular risk assessment: addition of CKD and race to the Framingham equation. Am Heart J. 2012;164(6):925-31.e2. |
| 88. | Dunder K, Lind L, Zethelius B, Berglund L, Lithell H. Evaluation of a scoring scheme, including proinsulin and the apolipoprotein B/apolipoprotein A1 ratio, for the risk of acute coronary events in middle-aged men: Uppsala Longitudinal Study of Adult Men (ULSAM). Am Heart J. 2004;148(4):596-601. |
| 89. | Duprez DA, Florea N, Zhong W, Grandits GA, Hawthorne CK, Hoke L, et al. Vascular and cardiac functional and structural screening to identify risk of future morbid events: preliminary observations. J Am Soc Hypertens. 2011;5(5):401-9. Epub 2011/07/02. |
| 90. | Dutta A, Henley W, Lang IA, Murray A, Guralnik J, Wallace RB, et al. The coronary artery disease-associated 9p21 variant and later life 20-year survival to cohort extinction. Circulation Cardiovascular Genetics. 2011;4(5):542-8. |
| 91. | Dutta A, Henley W, Pilling LC, Wallace RB, Melzer D. Uric acid measurement improves prediction of cardiovascular mortality in later life. J Am Geriatr Soc. 2013;61(3):319-26. |
| 92. | Emerging Risk Factors Collaboration, Di Angelantonio E, Gao P, Pennells L, Kaptoge S, Caslake M, et al. Lipid-related markers and cardiovascular disease prediction. JAMA. 2012;307(23):2499-506. |
| 93. | Empana JP, Ducimetiere P, Arveiler D, Ferrieres J, Evans A, Ruidavets JB, et al. Are the Framingham and PROCAM coronary heart disease risk functions applicable to different European populations? The PRIME Study. Eur Heart J. 2003;24(21):1903-11. |
| 94. | Empana JP, Tafflet M, Escolano S, Vergnaux AC, Bineau S, Ruidavets JB, et al. Predicting CHD risk in France: A pooled analysis of the D.E.S.I.R., Three City, PRIME, and SU.VI.MAX studies. Eur J Cardiovasc Prev Rehabil. 2011;18(2):175-85. |
| 95. | Erbel R, Mohlenkamp S, Lehmann N, Schmermund A, Moebus S, Stang A, et al. Sex related cardiovascular risk stratification based on quantification of atherosclerosis and inflammation. Atherosclerosis. 2008;197(2):662-72. Epub 2007/03/28. |
| 96. | Erbel R, Mohlenkamp S, Moebus S, Schmermund A, Lehmann N, Stang A, et al. Coronary risk stratification, discrimination, and reclassification improvement based on quantification of subclinical coronary atherosclerosis: the Heinz Nixdorf Recall study. J Am Coll Cardiol. 2010;56(17):1397-406. |
| 97. | Erikssen G, Bodegard J, Bjornholt JV, Liestol K, Thelle DS, Erikssen J. Exercise testing of healthy men in a new perspective: from diagnosis to prognosis. Eur Heart J. 2004;25(11):978-86. |
| 98. | Faeh D, Braun J, Rufibach K, Puhan MA, Marques-Vidal P, Bopp M. Population Specific and Up to Date Cardiovascular Risk Charts Can Be Efficiently Obtained with Record Linkage of Routine and Observational Data. PLoS ONE. 2013;8(2). |
| 99. | Ferrario M, Chiodini P, Chambless LE, Cesana G, Vanuzzo D, Panico S, et al. Prediction of coronary events in a low incidence population. Assessing accuracy of the CUORE Cohort Study prediction equation. Int J Epidemiol. 2005;34(2):413-21. |
| 100. | Fiscella K, Tancredi D, Franks P. Adding socioeconomic status to Framingham scoring to reduce disparities in coronary risk assessment. Am Heart J. 2009;157(6):988-94. |
| 101. | Folsom AR, Chambless LE, Duncan BB, Gilbert AC, Pankow JS, Atherosclerosis Risk in Communities Study I. Prediction of coronary heart disease in middle-aged adults with diabetes. Diabetes Care. 2003;26(10):2777-84. |
| 102. | Franks P, Tancredi DJ, Winters P, Fiscella K. Including socioeconomic status in coronary heart disease risk estimation. Ann Fam Med. 2010;8(5):447-53. |
| 103. | Friedland DR, Cederberg C, Tarima S. Audiometric pattern as a predictor of cardiovascular status: development of a model for assessment of risk. Laryngoscope. 2009;119(3):473-86. |
| 104. | Gaziano TA, Young CR, Fitzmaurice G, Atwood S, Gaziano JM. Laboratory-based versus non-laboratory-based method for assessment of cardiovascular disease risk: the NHANES I Follow-up Study cohort. Lancet. 2008;371(9616):923-31. |
| 105. | Glynn RJ, L'Italien GJ, Sesso HD, Jackson EA, Buring JE. Development of predictive models for long-term cardiovascular risk associated with systolic and diastolic blood pressure. Hypertension. 2002;39(1):105-10. Epub 2002/01/19. |
| 106. | Greenland P, LaBree L, Azen SP, Doherty TM, Detrano RC. Coronary artery calcium score combined with Framingham score for risk prediction in asymptomatic individuals.[Erratum appears in JAMA. 2004 Feb 4;291(5):563]. JAMA. 2004;291(2):210-5. |
| 107. | Gulati M, Arnsdorf MF, Shaw LJ, Pandey DK, Thisted RA, Lauderdale DS, et al. Prognostic value of the duke treadmill score in asymptomatic women. Am J Cardiol. 2005;96(3):369-75. |
| 108. | Hadaegh F, Mohebi R, Bozorgmanesh M, Saadat N, Sheikholeslami F, Azizi F. Electrocardiographic abnormalities improve classification of coronary heart disease risk in women: Tehran Lipid and Glucose Study. Atherosclerosis. 2012;222(1):110-5. |
| 109. | Haluska BA, Jeffries L, Carlier S, Marwick TH. Measurement of arterial distensibility and compliance to assess prognosis. Atherosclerosis. 2010;209(2):474-80. |
| 110. | Hamer M, Chida Y, Stamatakis E. Utility of C-reactive protein for cardiovascular risk stratification across three age groups in subjects without existing cardiovascular diseases. Am J Cardiol. 2009;104(4):538-42. |
| 111. | Hense HW, Schulte H, Lowel H, Assmann G, Keil U. Framingham risk function overestimates risk of coronary heart disease in men and women from Germany--results from the MONICA Augsburg and the PROCAM cohorts. Eur Heart J. 2003;24(10):937-45. Epub 2003/04/26. |
| 112. | Hense H-W, Koesters E, Wellmann J, Meisinger C, Volzke H, Keil U. Evaluation of a recalibrated Systematic Coronary Risk Evaluation cardiovascular risk chart: results from Systematic Coronary Risk Evaluation Germany. Eur J Cardiovasc Prev Rehabil. 2008;15(4):409-15. |
| 113. | Hippisley-Cox J, Coupland C, Robson J, Brindle P. Derivation, validation, and evaluation of a new QRISK model to estimate lifetime risk of cardiovascular disease: cohort study using QResearch database. BMJ. 2010;341:c6624. |
| 114. | Hippisley-Cox J, Coupland C, Vinogradova Y, Robson J, Brindle P. Performance of the QRISK cardiovascular risk prediction algorithm in an independent UK sample of patients from general practice: a validation study. Heart. 2008;94(1):34-9. |
| 115. | Hippisley-Cox J, Coupland C, Vinogradova Y, Robson J, May M, Brindle P. Derivation and validation of QRISK, a new cardiovascular disease risk score for the United Kingdom: prospective open cohort study. BMJ. 2007;335(7611):136. |
| 116. | Hippisley-Cox J, Coupland C, Vinogradova Y, Robson J, Minhas R, Sheikh A, et al. Predicting cardiovascular risk in England and Wales: prospective derivation and validation of QRISK2. BMJ. 2008;336(7659):1475-82. |
| 117. | Hoes AW, Grobbee DE, Valkenburg HA, Lubsen J, Hofman A. Cardiovascular risk and all-cause mortality; a 12 year follow-up study in The Netherlands. Eur J Epidemiol. 1993;9(3):285-92. Epub 1993/05/01. |
| 118. | Houterman S, Boshuizen HC, Verschuren WM, Giampaoli S, Nissinen A, Menotti A, et al. Predicting cardiovascular risk in the elderly in different European countries. Eur Heart J. 2002;23(4):294-300. Epub 2002/01/29. |
| 119. | Hsia J, Rodabough RJ, Manson JE, Liu S, Freiberg MS, Graettinger W, et al. Evaluation of the American Heart Association cardiovascular disease prevention guideline for women. Circ Cardiovasc Qual Outcomes. 2010;3(2):128-34. |
| 120. | Hughes MF, Saarela O, Blankenberg S, Zeller T, Havulinna AS, Kuulasmaa K, et al. A multiple biomarker risk score for guiding clinical decisions using a decision curve approach. Eur J Prev Cardiol. 2012;19(4):874-84. |
| 121. | Hughes MF, Saarela O, Stritzke J, Kee F, Silander K, Klopp N, et al. Genetic markers enhance coronary risk prediction in men: the MORGAM prospective cohorts. PLoS ONE. 2012;7(7):e40922. |
| 122. | Humphries SE, Cooper JA, Talmud PJ, Miller GJ. Candidate gene genotypes, along with conventional risk factor assessment, improve estimation of coronary heart disease risk in healthy UK men. Clin Chem. 2007;53(1):8-16. |
| 123. | Hurley LP, Dickinson LM, Estacio RO, Steiner JF, Havranek EP. Prediction of cardiovascular death in racial/ethnic minorities using Framingham risk factors. Circ Cardiovasc Qual Outcomes. 2010;3(2):181-7. |
| 124. | Iqbal FM, Al Jaroudi W, Sanam K, Sweeney A, Heo J, Iskandrian AE, et al. Reclassification of cardiovascular risk in patients with normal myocardial perfusion imaging using heart rate response to vasodilator stress. Am J Cardiol. 2013;111(2):190-5. |
| 125. | Ishikawa S, Matsumoto M, Kayaba K, Gotoh T, Nago N, Tsutsumi A, et al. Risk charts illustrating the 10-year risk of stroke among residents of Japanese rural communities: the JMS Cohort Study. J Epidemiol. 2009;19(2):101-6. |
| 126. | Ito H, Pacold IV, Durazo-Arvizu R, Liu K, Shilipak MG, Goff DC, Jr., et al. The effect of including cystatin C or creatinine in a cardiovascular risk model for asymptomatic individuals: the multi-ethnic study of atherosclerosis. Am J Epidemiol. 2011;174(8):949-57. |
| 127. | Jalal D, Chonchol M, Etgen T, Sander D. C-reactive protein as a predictor of cardiovascular events in elderly patients with chronic kidney disease. J Nephrol. 2012;25(5):719-25. |
| 128. | Janssen I, Katzmarzyk PT, Church TS, Blair SN. The Cooper Clinic Mortality Risk Index: clinical score sheet for men. Am J Prev Med. 2005;29(3):194-203. |
| 129. | Jimenez-Corona A, Lopez-Ridaura R, Williams K, Gonzalez-Villalpando ME, Simon J, Gonzalez-Villalpando C. Applicability of Framingham risk equations for studying a low-income Mexican population. Salud Publica Mex. 2009;51(4):298-305. |
| 130. | Kamstrup PR, Tybjaerg-Hansen A, Nordestgaard BG. Extreme lipoprotein(a) levels and improved cardiovascular risk prediction. J Am Coll Cardiol. 2013;61(11):1146-56. |
| 131. | Kang HM, Kim D-J. Metabolic Syndrome versus Framingham Risk Score for Association of Self-Reported Coronary Heart Disease: The 2005 Korean Health and Nutrition Examination Survey. Diabetes Metab J. 2012;36(3):237-44. |
| 132. | Kannel WB, McGee D, Gordon T. A general cardiovascular risk profile: the Framingham Study. Am J Cardiol. 1976;38(1):46-51. Epub 1976/07/01. |
| 133. | Kathiresan S, Melander O, Anevski D, Guiducci C, Burtt NP, Roos C, et al. Polymorphisms associated with cholesterol and risk of cardiovascular events. N Engl J Med. 2008;358(12):1240-9. |
| 134. | Katz D, Foxman B. How well do prediction equations predict? Using receiver operating characteristic curves and accuracy curves to compare validity and generalizability. Epidemiology. 1993;4(4):319-26. Epub 1993/07/01. |
| 135. | Ketola E, Laatikainen T, Vartiainen E. Evaluating risk for cardiovascular diseases--vain or value? How do different cardiovascular risk scores act in real life. Eur J Public Health. 2010;20(1):107-12. |
| 136. | Keys A, Aravanis C, Blackburn H, Van Buchem FS, Buzina R, Djordjevic BS, et al. Probability of middle-aged men developing coronary heart disease in five years. Circulation. 1972;45(4):815-28. Epub 1972/04/01. |
| 137. | Khalili D, Hadaegh F, Soori H, Steyerberg EW, Bozorgmanesh M, Azizi F. Clinical usefulness of the Framingham cardiovascular risk profile beyond its statistical performance: the Tehran Lipid and Glucose Study. Am J Epidemiol. 2012;176(3):177-86. |
| 138. | Knuiman MW, Vu HT. Prediction of coronary heart disease mortality in Busselton, Western Australia: an evaluation of the Framingham, national health epidemiologic follow up study, and WHO ERICA risk scores. J Epidemiol Community Health. 1997;51(5):515-9. Epub 1998/01/13. |
| 139. | Knuiman MW, Vu HT, Bartholomew HC. Multivariate risk estimation for coronary heart disease: the Busselton Health Study. Aust N Z J Public Health. 1998;22(7):747-53. Epub 1999/01/16. |
| 140. | Koizumi J, Shimizu M, Miyamoto S, Takeda R, Ohka T, Kanaya H, et al. Risk evaluation of coronary heart disease and cerebrovascular disease by the Japan Atherosclerosis Society Guidelines 2002 using the cohort of the Holicos-PAT study. J Atheroscler Thromb. 2005;12(1):48-52. |
| 141. | Koller MT, Leening MJG, Wolbers M, Steyerberg EW, Hunink MGM, Schoop R, et al. Development and validation of a coronary risk prediction model for older U.S. and European persons in the cardiovascular health study and the Rotterdam Study. Ann Intern Med. 2012;157(6):389-97. |
| 142. | Koller MT, Steyerberg EW, Wolbers M, Stijnen T, Bucher HC, Hunink MGM, et al. Validity of the Framingham point scores in the elderly: results from the Rotterdam study. Am Heart J. 2007;154(1):87-93. |
| 143. | Larson MG. Assessment of cardiovascular risk factors in the elderly: the Framingham Heart Study. Stat Med. 1995;14(16):1745-56. Epub 1995/08/30. |
| 144. | Laurier D, Nguyen PC, Cazelles B, Segond P. Estimation of CHD risk in a French working population using a modified Framingham model. The PCV-METRA Group. J Clin Epidemiol. 1994;47(12):1353-64. Epub 1994/12/01. |
| 145. | Leaverton PE, Sorlie PD, Kleinman JC, Dannenberg AL, Ingster-Moore L, Kannel WB, et al. Representativeness of the Framingham risk model for coronary heart disease mortality: a comparison with a national cohort study. J Chronic Dis. 1987;40(8):775-84. Epub 1987/01/01. |
| 146. | Lee ET, Howard BV, Wang W, Welty TK, Galloway JM, Best LG, et al. Prediction of coronary heart disease in a population with high prevalence of diabetes and albuminuria: the Strong Heart Study. Circulation. 2006;113(25):2897-905. |
| 147. | Lee J, Heng D, Ma S, Chew S-K, Hughes K, Tai ES. The metabolic syndrome and mortality: the Singapore Cardiovascular Cohort Study. Clin Endocrinol (Oxf). 2008;69(2):225-30. |
| 148. | Levy D, Wilson PW, Anderson KM, Castelli WP. Stratifying the patient at risk from coronary disease: new insights from the Framingham Heart Study. Am Heart J. 1990;119(3 Pt 2):712-7; discussion 7. Epub 1990/03/01. |
| 149. | Lindman AS, Veierod MB, Pedersen JI, Tverdal A, Njolstad I, Selmer R. The ability of the SCORE high-risk model to predict 10-year cardiovascular disease mortality in Norway. Eur J Cardiovasc Prev Rehabil. 2007;14(4):501-7. |
| 150. | L'Italien G, Ford I, Norrie J, LaPuerta P, Ehreth J, Jackson J, et al. The cardiovascular event reduction tool (CERT)--a simplified cardiac risk prediction model developed from the West of Scotland Coronary Prevention Study (WOSCOPS). Am J Cardiol. 2000;85(6):720-4. Epub 2002/05/10. |
| 151. | Liu J, Hong Y, D'Agostino RB, Sr., Wu Z, Wang W, Sun J, et al. Predictive value for the Chinese population of the Framingham CHD risk assessment tool compared with the Chinese Multi-Provincial Cohort Study. JAMA. 2004;291(21):2591-9. |
| 152. | Lloyd-Jones DM, Wilson PWF, Larson MG, Beiser A, Leip EP, D'Agostino RB, et al. Framingham risk score and prediction of lifetime risk for coronary heart disease. Am J Cardiol. 2004;94(1):20-4. |
| 153. | Lumley T, Kronmal RA, Cushman M, Manolio TA, Goldstein S. A stroke prediction score in the elderly: validation and Web-based application. J Clin Epidemiol. 2002;55(2):129-36. Epub 2002/01/26. |
| 154. | Macfarlane PW, Norrie J. The value of the electrocardiogram in risk assessment in primary prevention: Experience from the West of Scotland Coronary Prevention Study. J Electrocardiol. 2007;40(1):101-9. |
| 155. | Mainous AG, 3rd, Everett CJ, Player MS, King DE, Diaz VA. Importance of a patient's personal health history on assessments of future risk of coronary heart disease. J Am Board Fam Med. 2008;21(5):408-13. |
| 156. | Mainous AG, 3rd, Koopman RJ, Diaz VA, Everett CJ, Wilson PWF, Tilley BC. A coronary heart disease risk score based on patient-reported information. Am J Cardiol. 2007;99(9):1236-41. |
| 157. | Manickam P, Rathod A, Panaich S, Hari P, Veeranna V, Badheka A, et al. Comparative prognostic utility of conventional and novel lipid parameters for cardiovascular disease risk prediction: do novel lipid parameters offer an advantage? J Clin Lipidol. 2011;5(2):82-90. |
| 158. | Mannan H, Stevenson C, Peeters A, Walls H, McNeil J. Framingham risk prediction equations for incidence of cardiovascular disease using detailed measures for smoking. Heart Int. 2010;5(2):e11. |
| 159. | Mannan HR, Stevenson CE, Peeters A, McNeil JJ. A new set of risk equations for predicting long term risk of all-cause mortality using cardiovascular risk factors. Prev Med. 2013;56(1):41-5. |
| 160. | Mannan HR, Stevenson CE, Peeters A, Walls HL, McNeil JJ. Age at quitting smoking as a predictor of risk of cardiovascular disease incidence independent of smoking status, time since quitting and pack-years. BMC Research Notes. 2011;4:39. |
| 161. | Marrugat J, D'Agostino R, Sullivan L, Elosua R, Wilson P, Ordovas J, et al. An adaptation of the Framingham coronary heart disease risk function to European Mediterranean areas. J Epidemiol Community Health. 2003;57(8):634-8. Epub 2003/07/29. |
| 162. | Marrugat J, Solanas P, D'Agostino R, Sullivan L, Ordovas J, Cordon F, et al. Coronary risk estimation in Spain using a calibrated Framingham function. Rev Esp Cardiol. 2003;56(3):253-61. Epub 2003/03/08. Estimacion del riesgo coronario en Espana mediante la ecuacion de Framingham calibrada. |
| 163. | Marrugat J, Subirana I, Comin E, Cabezas C, Vila J, Elosua R, et al. Validity of an adaptation of the Framingham cardiovascular risk function: The VERIFICA study. J Epidemiol Community Health. 2007;61(1):40-7. |
| 164. | Matsumoto M, Ishikawa S, Kayaba K, Gotoh T, Nago N, Tsutsumi A, et al. Risk charts illustrating the 10-year risk of myocardial infarction among residents of Japanese rural communities: the JMS Cohort Study. J Epidemiol. 2009;19(2):94-100. |
| 165. | May M, Lawlor DA, Brindle P, Patel R, Ebrahim S. Cardiovascular disease risk assessment in older women: can we improve on Framingham? British Women's Heart and Health prospective cohort study. Heart. 2006;92(10):1396-401. |
| 166. | May M, Sterne JAC, Shipley M, Brunner E, d'Agostino R, Whincup P, et al. A coronary heart disease risk model for predicting the effect of potent antiretroviral therapy in HIV-1 infected men. Int J Epidemiol. 2007;36(6):1309-18. |
| 167. | McGeechan K, Liew G, Macaskill P, Irwig L, Klein R, Sharrett AR, et al. Risk prediction of coronary heart disease based on retinal vascular caliber (from the Atherosclerosis Risk In Communities [ARIC] Study). Am J Cardiol. 2008;102(1):58-63. |
| 168. | McGorrian C, Yusuf S, Islam S, Jung H, Rangarajan S, Avezum A, et al. Estimating modifiable coronary heart disease risk in multiple regions of the world: the INTERHEART Modifiable Risk Score. Eur Heart J. 2011;32(5):581-9. |
| 169. | McNeil JJ, Peeters A, Liew D, Lim S, Vos T. A model for predicting the future incidence of coronary heart disease within percentiles of coronary heart disease risk. J Cardiovasc Risk. 2001;8(1):31-7. Epub 2001/03/10. |
| 170. | Meigs JB, Nathan DM, D'Agostino Sr RB, Wilson PWF. Fasting and postchallenge glycemia and cardiovascular disease risk: The framingham offspring study. Diabetes Care. 2002;25(10):1845-50. |
| 171. | Melander O, Newton-Cheh C, Almgren P, Hedblad B, Berglund G, Engstrom G, et al. Novel and conventional biomarkers for prediction of incident cardiovascular events in the community. JAMA. 2009;302(1):49-57. |
| 172. | Menotti A, Farchi G, Seccareccia F. The prediction of coronary heart disease mortality as a function of major risk factors in over 30 000 men in the Italian RIFLE pooling Project. A comparison with the MRFIT primary screenees. The RIFLE research group. J Cardiovasc Risk. 1994;1(3):263-70. Epub 1994/10/01. |
| 173. | Menotti A, Keys A, Kromhout D, Nissinen A, Blackburn H, Fidanza F, et al. Twenty-five-year mortality from coronary heart disease and its prediction in five cohorts of middle-aged men in Finland, The Netherlands, and Italy. Prev Med. 1990;19(3):270-8. Epub 1990/05/01. |
| 174. | Menotti A, Lanti M, Agabiti-Rosei E, Carratelli L, Cavera G, Dormi A, et al. Riskard 2005. New tools for prediction of cardiovascular disease risk derived from Italian population studies. Nutr Metab Cardiovasc Dis. 2005;15(6):426-40. |
| 175. | Menotti A, Lanti M, Puddu PE, Carratelli L, Mancini M, Motolese M, et al. The risk functions incorporated in Riscard 2002: a software for the prediction of cardiovascular risk in the general population based on Italian data. Ital Heart J. 2002;3(2):114-21. Epub 2002/04/03. |
| 176. | Menotti A, Lanti M, Puddu PE, Mancini M, Zanchetti A, Cirillo M, et al. First risk functions for prediction of coronary and cardiovascular disease incidence in the Gubbio Population Study. Ital Heart J. 2000;1(6):394-9. Epub 2000/08/10. |
| 177. | Merry AHH, Boer JMA, Schouten LJ, Ambergen T, Steyerberg EW, Feskens EJM, et al. Risk prediction of incident coronary heart disease in The Netherlands: re-estimation and improvement of the SCORE risk function. Eur J Prev Cardiol. 2012;19(4):840-8. |
| 178. | Milne R, Gamble G, Whitlock G, Jackson R. Discriminative ability of a risk-prediction tool derived from the Framingham Heart Study compared with single risk factors. N Z Med J. 2003;116(1185):U663. |
| 179. | Milne R, Gamble G, Whitlock G, Jackson R. Framingham Heart Study risk equation predicts first cardiovascular event rates in New Zealanders at the population level. N Z Med J. 2003;116(1185):U662. Epub 2003/11/15. |
| 180. | Mitchell GF, Hwang S-J, Vasan RS, Larson MG, Pencina MJ, Hamburg NM, et al. Arterial stiffness and cardiovascular events: the Framingham Heart Study. Circulation. 2010;121(4):505-11. |
| 181. | Mohammadreza B, Farzad H, Davoud K, Fereidoun Prof AF. Prognostic significance of the complex "Visceral Adiposity Index" vs. simple anthropometric measures: Tehran lipid and glucose study. Cardiovasc Diabetol. 2012;11:20. |
| 182. | Mohlenkamp S, Lehmann N, Greenland P, Moebus S, Kalsch H, Schmermund A, et al. Coronary artery calcium score improves cardiovascular risk prediction in persons without indication for statin therapy. Atherosclerosis. 2011;215(1):229-36. |
| 183. | Mohlenkamp S, Lehmann N, Moebus S, Schmermund A, Dragano N, Stang A, et al. Quantification of coronary atherosclerosis and inflammation to predict coronary events and all-cause mortality. J Am Coll Cardiol. 2011;57(13):1455-64. |
| 184. | Moons KG, Bots ML, Salonen JT, Elwood PC, Freire de Concalves A, Nikitin Y, et al. Prediction of stroke in the general population in Europe (EUROSTROKE): Is there a role for fibrinogen and electrocardiography? J Epidemiol Community Health. 2002;56 Suppl 1:i30-6. Epub 2002/01/30. |
| 185. | Mora S, Redberg RF, Sharrett AR, Blumenthal RS. Enhanced risk assessment in asymptomatic individuals with exercise testing and Framingham risk scores. Circulation. 2005;112(11):1566-72. |
| 186. | Morrison AC, Bare LA, Chambless LE, Ellis SG, Malloy M, Kane JP, et al. Prediction of coronary heart disease risk using a genetic risk score: the Atherosclerosis Risk in Communities Study. Am J Epidemiol. 2007;166(1):28-35. |
| 187. | Munir JA, Wu H, Bauer K, Bindeman J, Byrd C, O'Malley P, et al. Impact of coronary calcium on arterial age and coronary heart disease risk estimation using the MESA arterial age calculator. Atherosclerosis. 2010;211(2):467-70. Epub 2010/04/10. |
| 188. | Murphy TP, Dhangana R, Pencina MJ, D'Agostino RB, Sr. Ankle-brachial index and cardiovascular risk prediction: an analysis of 11,594 individuals with 10-year follow-up. Atherosclerosis. 2012;220(1):160-7. |
| 189. | Murphy TP, Dhangana R, Pencina MJ, Zafar AM, D'Agostino RB. Performance of current guidelines for coronary heart disease prevention: optimal use of the Framingham-based risk assessment. Atherosclerosis. 2011;216(2):452-7. |
| 190. | Nambi V, Boerwinkle E, Lawson K, Brautbar A, Chambless L, Franeschini N, et al. The 9p21 genetic variant is additive to carotid intima media thickness and plaque in improving coronary heart disease risk prediction in white participants of the Atherosclerosis Risk in Communities (ARIC) Study. Atherosclerosis. 2012;222(1):135-7. |
| 191. | Nambi V, Chambless L, Folsom AR, He M, Hu Y, Mosley T, et al. Carotid intima-media thickness and presence or absence of plaque improves prediction of coronary heart disease risk: the ARIC (Atherosclerosis Risk In Communities) study. J Am Coll Cardiol. 2010;55(15):1600-7. |
| 192. | Nambi V, Chambless L, He M, Folsom AR, Mosley T, Boerwinkle E, et al. Common carotid artery intima-media thickness is as good as carotid intima-media thickness of all carotid artery segments in improving prediction of coronary heart disease risk in the Atherosclerosis Risk in Communities (ARIC) study. Eur Heart J. 2012;33(2):183-90. |
| 193. | Nelson MR, Ramsay E, Ryan P, Willson K, Tonkin AM, Wing L, et al. A score for the prediction of cardiovascular events in the hypertensive aged. Am J Hypertens. 2012;25(2):190-4. |
| 194. | Nelson MR, Ryan P, Tonkin AM, Ramsay E, Willson K, Wing LWH, et al. Prediction of cardiovascular events in subjects in the second Australian National Blood Pressure study. Hypertension. 2010;56(1):44-8. |
| 195. | Nielsen M, Ganz M, Lauze F, Pettersen PC, de Bruijne M, Clarkson TB, et al. Distribution, size, shape, growth potential and extent of abdominal aortic calcified deposits predict mortality in postmenopausal women. BMC Cardiovasc Disord. 2010;10:56. |
| 196. | Nippon Data Research Group. Risk assessment chart for death from cardiovascular disease based on a 19-year follow-up study of a Japanese representative population. Circ J. 2006;70(10):1249-55. |
| 197. | Noda H, Maruyama K, Iso H, Dohi S, Terai T, Fujioka S, et al. Prediction of myocardial infarction using coronary risk scores among Japanese male workers: 3M Study. J Atheroscler Thromb. 2010;17(5):452-9. |
| 198. | Nordestgaard BG, Adourian AS, Freiberg JJ, Guo Y, Muntendam P, Falk E. Risk factors for near-term myocardial infarction in apparently healthy men and women. Clin Chem. 2010;56(4):559-67. |
| 199. | Novo S, Visconti CL, Amoroso GR, Corrado E, Fazio G, Muratori I, et al. Asymptomatic carotid lesions add to cardiovascular risk prediction. Eur J Cardiovasc Prev Rehabil. 2010;17(5):514-8. |
| 200. | Nozaki T, Sugiyama S, Koga H, Sugamura K, Ohba K, Matsuzawa Y, et al. Significance of a multiple biomarkers strategy including endothelial dysfunction to improve risk stratification for cardiovascular events in patients at high risk for coronary heart disease. J Am Coll Cardiol. 2009;54(7):601-8. |
| 201. | Odell PM, Anderson KM, Kannel WB. New models for predicting cardiovascular events. J Clin Epidemiol. 1994;47(6):583-92. Epub 1994/06/01. |
| 202. | Oksala N, Seppala I, Hernesniemi J, Lyytikainen L-P, Kahonen M, Makela K-M, et al. Complementary prediction of cardiovascular events by estimated apo- and lipoprotein concentrations in the working age population. The Health 2000 Study. Ann Med. 2013;45(2):141-8. |
| 203. | Olsen MH, Wachtell K, Ibsen H, Lindholm L, Kjeldsen SE, Omvik P, et al. Changes in subclinical organ damage vs. in Framingham risk score for assessing cardiovascular risk reduction during continued antihypertensive treatment: a LIFE substudy. J Hypertens. 2011;29(5):997-1004. |
| 204. | Onat A, Can G, Hergenc G, Ugur M, Yuksel H. Coronary disease risk prediction algorithm warranting incorporation of C-reactive protein in Turkish adults, manifesting sex difference. Nutr Metab Cardiovasc Dis. 2012;22(8):643-50. |
| 205. | Orford JL, Sesso HD, Stedman M, Gagnon D, Vokonas P, Gaziano JM. A comparison of the Framingham and European Society of Cardiology coronary heart disease risk prediction models in the normative aging study. Am Heart J. 2002;144(1):95-100. Epub 2002/07/03. |
| 206. | Panagiotakos DB, Fitzgerald AP, Pitsavos C, Pipilis A, Graham I, Stefanadis C. Statistical modelling of 10-year fatal cardiovascular disease risk in Greece: the HellenicSCORE (a calibration of the ESC SCORE project). Hellenic J Cardiol. 2007;48(2):55-63. |
| 207. | Panagiotakos DB, Pitsavos C, Stefanadis C. Inclusion of dietary evaluation in cardiovascular disease risk prediction models increases accuracy and reduces bias of the estimations. Risk Anal. 2009;29(2):176-86. |
| 208. | Pandya A, Weinstein MC, Gaziano TA. A comparative assessment of non-laboratory-based versus commonly used laboratory-based cardiovascular disease risk scores in the NHANES III population. PLoS ONE. 2011;6(5):e20416. |
| 209. | Park Y, Lim J, Lee J, Kim SG. Erythrocyte fatty acid profiles can predict acute non-fatal myocardial infarction. Br J Nutr. 2009;102(9):1355-61. Epub 2009/06/10. |
| 210. | Paynter NP, Chasman DI, Buring JE, Shiffman D, Cook NR, Ridker PM. Cardiovascular disease risk prediction with and without knowledge of genetic variation at chromosome 9p21.3. Ann Intern Med. 2009;150(2):65-72. |
| 211. | Paynter NP, Chasman DI, Pare G, Buring JE, Cook NR, Miletich JP, et al. Association between a literature-based genetic risk score and cardiovascular events in women. JAMA. 2010;303(7):631-7. Epub 2010/02/18. |
| 212. | Paynter NP, Mazer NA, Pradhan AD, Gaziano JM, Ridker PM, Cook NR. Cardiovascular risk prediction in diabetic men and women using hemoglobin A1c vs diabetes as a high-risk equivalent. Arch Intern Med. 2011;171(19):1712-8. |
| 213. | Pencina MJ, D'Agostino RB, Sr., Larson MG, Massaro JM, Vasan RS. Predicting the 30-year risk of cardiovascular disease: the framingham heart study. Circulation. 2009;119(24):3078-84. |
| 214. | Petersson U, Ostgren CJ, Brudin L, Nilsson PM. A consultation-based method is equal to SCORE and an extensive laboratory-based method in predicting risk of future cardiovascular disease. Eur J Cardiovasc Prev Rehabil. 2009;16(5):536-40. |
| 215. | Plichart M, Celermajer DS, Zureik M, Helmer C, Jouven X, Ritchie K, et al. Carotid intima-media thickness in plaque-free site, carotid plaques and coronary heart disease risk prediction in older adults. The Three-City Study. Atherosclerosis. 2011;219(2):917-24. |
| 216. | Pocock SJ, McCormack V, Gueyffier F, Boutitie F, Fagard RH, Boissel JP. A score for predicting risk of death from cardiovascular disease in adults with raised blood pressure, based on individual patient data from randomised controlled trials. BMJ. 2001;323(7304):75-81. Epub 2001/07/14. |
| 217. | Poels MMF, Steyerberg EW, Wieberdink RG, Hofman A, Koudstaal PJ, Ikram MA, et al. Assessment of cerebral small vessel disease predicts individual stroke risk. J Neurol Neurosurg Psychiatry. 2012;83(12):1174-9. |
| 218. | Polak JF, Pencina MJ, Pencina KM, O'Donnell CJ, Wolf PA, D'Agostino RB, Sr. Carotid-wall intima-media thickness and cardiovascular events. N Engl J Med. 2011;365(3):213-21. |
| 219. | Polonsky TS, McClelland RL, Jorgensen NW, Bild DE, Burke GL, Guerci AD, et al. Coronary artery calcium score and risk classification for coronary heart disease prediction. JAMA. 2010;303(16):1610-6. |
| 220. | Prati P, Tosetto A, Casaroli M, Bignamini A, Canciani L, Bornstein N, et al. Carotid plaque morphology improves stroke risk prediction: usefulness of a new ultrasonographic score. Cerebrovasc Dis. 2011;31(3):300-4. |
| 221. | Prugger C, Luc G, Haas B, Arveiler D, Machez E, Ferrieres J, et al. Adipocytokines and the risk of ischemic stroke: the PRIME Study. Ann Neurol. 2012;71(4):478-86. |
| 222. | Qiao Q, Gao W, Laatikainen T, Vartiainen E. Layperson-oriented vs. clinical-based models for prediction of incidence of ischemic stroke: National FINRISK Study. Int J Stroke. 2012;7(8):662-8. |
| 223. | Rachas A, Raffaitin C, Barberger-Gateau P, Helmer C, Ritchie K, Tzourio C, et al. Clinical usefulness of the metabolic syndrome for the risk of coronary heart disease does not exceed the sum of its individual components in older men and women. The Three-City (3C) Study. Heart. 2012;98(8):650-5. |
| 224. | Ramachandran S, French JM, Vanderpump MP, Croft P, Neary RH. Using the Framingham model to predict heart disease in the United Kingdom: retrospective study. BMJ. 2000;320(7236):676-7. Epub 2000/03/11. |
| 225. | Ramsay SE, Morris RW, Whincup PH, Papacosta AO, Thomas MC, Wannamethee SG. Prediction of coronary heart disease risk by Framingham and SCORE risk assessments varies by socioeconomic position: results from a study in British men. Eur J Cardiovasc Prev Rehabil. 2011;18(2):186-93. |
| 226. | Rana JS, Cote M, Despres JP, Sandhu MS, Talmud PJ, Ninio E, et al. Inflammatory biomarkers and the prediction of coronary events among people at intermediate risk: the EPIC-Norfolk prospective population study. Heart. 2009;95(20):1682-7. |
| 227. | Reissigova J, Zvarova J. The Framingham risk function underestimated absolute coronary heart disease risk in Czech men. Methods Inf Med. 2007;46(1):43-9. |
| 228. | Riddell T, Wells S, Jackson R, Lee A-W, Crengle S, Bramley D, et al. Performance of Framingham cardiovascular risk scores by ethnic groups in New Zealand: PREDICT CVD-10. N Z Med J. 2010;123(1309):50-61. |
| 229. | Ridker PM, Buring JE, Rifai N, Cook NR. Development and validation of improved algorithms for the assessment of global cardiovascular risk in women: the Reynolds Risk Score. JAMA. 2007;297(6):611-9. |
| 230. | Ridker PM, Paynter NP, Rifai N, Gaziano JM, Cook NR. C-reactive protein and parental history improve global cardiovascular risk prediction: the Reynolds Risk Score for men. Circulation. 2008;118(22):2243-51, 4p following 51. |
| 231. | Rifkin DE, Ix JH, Wassel CL, Criqui MH, Allison MA. Renal artery calcification and mortality among clinically asymptomatic adults. J Am Coll Cardiol. 2012;60(12):1079-85. |
| 232. | Rodondi N, Locatelli I, Aujesky D, Butler J, Vittinghoff E, Simonsick E, et al. Framingham risk score and alternatives for prediction of coronary heart disease in older adults. PLoS ONE. 2012;7(3):e34287. |
| 233. | Root M, Smith T. Prescribe by risk: the utility of a biomarker-based risk calculation in disease management to prevent heart disease. Dis Manag. 2005;8(2):106-13. |
| 234. | Rutten JHW, Mattace-Raso FUS, Steyerberg EW, Lindemans J, Hofman A, Wieberdink RG, et al. Amino-terminal pro-B-type natriuretic peptide improves cardiovascular and cerebrovascular risk prediction in the population: the Rotterdam study. Hypertension. 2010;55(3):785-91. |
| 235. | Ruwald MH, Ruwald AC, Jons C, Lamberts M, Hansen ML, Vinther M, et al. Evaluation of the chads2 risk score on short- and long-term all-cause and cardiovascular mortality after syncope. Clin Cardiol. 2013;36(5):262-8. |
| 236. | Sacco RL, Khatri M, Rundek T, Xu Q, Gardener H, Boden-Albala B, et al. Improving global vascular risk prediction with behavioral and anthropometric factors. The multiethnic NOMAS (Northern Manhattan Cohort Study). J Am Coll Cardiol. 2009;54(24):2303-11. |
| 237. | Saidj M, Jorgensen T, Prescott E, Borglykke A. Poor predictive ability of the risk chart SCORE in a Danish population. Dan Med J. 2013;60(5). |
| 238. | Saunders JT, Nambi V, de Lemos JA, Chambless LE, Virani SS, Boerwinkle E, et al. Cardiac troponin T measured by a highly sensitive assay predicts coronary heart disease, heart failure, and mortality in the Atherosclerosis Risk in Communities Study. Circulation. 2011;123(13):1367-76. |
| 239. | Scheltens T, Verschuren WMM, Boshuizen HC, Hoes AW, Zuithoff NP, Bots ML, et al. Estimation of cardiovascular risk: a comparison between the Framingham and the SCORE model in people under 60 years of age. Eur J Cardiovasc Prev Rehabil. 2008;15(5):562-6. |
| 240. | Schnabel RB, Sullivan LM, Levy D, Pencina MJ, Massaro JM, D'Agostino RB, Sr., et al. Development of a risk score for atrial fibrillation (Framingham Heart Study): a community-based cohort study. Lancet. 2009;373(9665):739-45. Epub 2009/03/03. |
| 241. | Schottker B, Muller H, Rothenbacher D, Brenner H. Fasting plasma glucose and HbA1c in cardiovascular risk prediction: A sex-specific comparison in individuals without diabetes mellitus. Diabetologia. 2013;56(1):92-100. |
| 242. | Sehestedt T, Jeppesen J, Hansen TW, Wachtell K, Ibsen H, Torp-Petersen C, et al. Risk prediction is improved by adding markers of subclinical organ damage to SCORE. Eur Heart J. 2010;31(7):883-91. |
| 243. | Sever PS, Poulter NR, Chang CL, Hingorani A, Thom SA, Hughes AD, et al. Evaluation of C-reactive protein prior to and on-treatment as a predictor of benefit from atorvastatin: observations from the Anglo-Scandinavian Cardiac Outcomes Trial. Eur Heart J. 2012;33(4):486-94. Epub 2011/07/30. |
| 244. | Shah S, Casas JP, Gaunt TR, Cooper J, Drenos F, Zabaneh D, et al. Influence of common genetic variation on blood lipid levels, cardiovascular risk, and coronary events in two British prospective cohort studies. Eur Heart J. 2013;34(13):972-81. |
| 245. | Shaper AG, Pocock SJ, Phillips AN, Walker M. Identifying men at high risk of heart attacks: strategy for use in general practice. Br Med J (Clin Res Ed). 1986;293(6545):474-9. Epub 1986/08/23. |
| 246. | Shara NM, Wang H, Valaitis E, Pehlivanova M, Carter EA, Resnick HE, et al. Comparison of estimated glomerular filtration rates and albuminuria in predicting risk of coronary heart disease in a population with high prevalence of diabetes mellitus and renal disease. Am J Cardiol. 2011;107(3):399-405. |
| 247. | Simmons RK, Coleman RL, Price HC, Holman RR, Khaw K-T, Wareham NJ, et al. Performance of the UK Prospective Diabetes Study Risk Engine and the Framingham Risk Equations in Estimating Cardiovascular Disease in the EPIC- Norfolk Cohort. Diabetes Care. 2009;32(4):708-13. |
| 248. | Simmons RK, Sharp S, Boekholdt SM, Sargeant LA, Khaw K-T, Wareham NJ, et al. Evaluation of the Framingham risk score in the European Prospective Investigation of Cancer-Norfolk cohort: does adding glycated hemoglobin improve the prediction of coronary heart disease events? Arch Intern Med. 2008;168(11):1209-16. |
| 249. | Simons LA, Simons J, Friedlander Y, McCallum J, Palaniappan L. Risk functions for prediction of cardiovascular disease in elderly Australians: the Dubbo Study. Med J Aust. 2003;178(3):113-6. Epub 2003/02/01. |
| 250. | Sivapalaratnam S, Boekholdt SM, Trip MD, Sandhu MS, Luben R, Kastelein JJP, et al. Family history of premature coronary heart disease and risk prediction in the EPIC-Norfolk prospective population study. Heart. 2010;96(24):1985-9. |
| 251. | Smink PA, Lambers Heerspink HJ, Gansevoort RT, de Jong PE, Hillege HL, Bakker SJL, et al. Albuminuria, estimated GFR, traditional risk factors, and incident cardiovascular disease: the PREVEND (Prevention of Renal and Vascular Endstage Disease) study. Am J Kidney Dis. 2012;60(5):804-11. |
| 252. | Smith JG, Newton-Cheh C, Almgren P, Struck J, Morgenthaler NG, Bergmann A, et al. Assessment of conventional cardiovascular risk factors and multiple biomarkers for the prediction of incident heart failure and atrial fibrillation. J Am Coll Cardiol. 2010;56(21):1712-9. |
| 253. | Stein PK, Barzilay JI. Relationship of abnormal heart rate turbulence and elevated CRP to cardiac mortality in low, intermediate, and high-risk older adults. J Cardiovasc Electrophysiol. 2011;22(2):122-7. |
| 254. | Stenlund H, Lonnberg G, Jenkins P, Norberg M, Persson M, Messner T, et al. Fewer deaths from cardiovascular disease than expected from the Systematic Coronary Risk Evaluation chart in a Swedish population. Eur J Cardiovasc Prev Rehabil. 2009;16(3):321-4. |
| 255. | Stern MP, Williams K, Gonzalez-Villalpando C, Hunt KJ, Haffner SM. Does the metabolic syndrome improve identification of individuals at risk of type 2 diabetes and/or cardiovascular disease? Diabetes Care. 2004;27(11):2676-81. |
| 256. | Stork S, Feelders RA, van den Beld AW, Steyerberg EW, Savelkoul HFJ, Lamberts SWJ, et al. Prediction of mortality risk in the elderly. Am J Med. 2006;119(6):519-25. |
| 257. | Suka M, Sugimori H, Yoshida K. Application of the updated Framingham risk score to Japanese men. Hypertens Res. 2001;24(6):685-9. Epub 2002/01/05. |
| 258. | Talmud PJ, Cooper JA, Palmen J, Lovering R, Drenos F, Hingorani AD, et al. Chromosome 9p21.3 coronary heart disease locus genotype and prospective risk of CHD in healthy middle-aged men. Clin Chem. 2008;54(3):467-74. |
| 259. | Tanabe N, Iso H, Okada K, Nakamura Y, Harada A, Ohashi Y, et al. Serum total and non-high-density lipoprotein cholesterol and the risk prediction of cardiovascular events - the JALS-ECC. Circ J. 2010;74(7):1346-56. Epub 2010/06/08. |
| 260. | Teramoto T, Ohashi Y, Nakaya N, Yokoyama S, Mizuno K, Nakamura H, et al. Practical risk prediction tools for coronary heart disease in mild to moderate hypercholesterolemia in Japan: originated from the MEGA study data. Circ J. 2008;72(10):1569-75. |
| 261. | Thanassoulis G, Peloso GM, Pencina MJ, Hoffmann U, Fox CS, Cupples LA, et al. A genetic risk score is associated with incident cardiovascular disease and coronary artery calcium the framingham heart study. Circ Cardiovasc Genet. 2012;5(1):113-21. |
| 262. | Thomsen TF, Davidsen M, Ibsen H, Jorgensen T, Jensen G, Borch-Johnsen K. A new method for CHD prediction and prevention based on regional risk scores and randomized clinical trials; PRECARD and the Copenhagen Risk Score. J Cardiovasc Risk. 2001;8(5):291-7. Epub 2001/11/10. |
| 263. | Thorsen RD, Jacobs DR, Jr., Grimm RH, Jr., Keys A, Taylor H, Blackburn H. Preventive cardiology in practice: a device for risk estimation and counseling in coronary disease. Prev Med. 1979;8(5):548-56. Epub 1979/09/01. |
| 264. | Tohidi M, Hadaegh F, Harati H, Azizi F. C-reactive protein in risk prediction of cardiovascular outcomes: Tehran Lipid and Glucose Study. Int J Cardiol. 2009;132(3):369-74. |
| 265. | Truelsen T, Lindenstrom E, Boysen G. Comparison of probability of stroke between the Copenhagen City Heart Study and the Framingham Study. Stroke. 1994;25(4):802-7. Epub 1994/04/01. |
| 266. | Truett J, Cornfield J, Kannel W. A multivariate analysis of the risk of coronary heart disease in Framingham. J Chronic Dis. 1967;20(7):511-24. Epub 1967/07/01. |
| 267. | Tsang TS, Barnes ME, Gersh BJ, Takemoto Y, Rosales AG, Bailey KR, et al. Prediction of risk for first age-related cardiovascular events in an elderly population: the incremental value of echocardiography. J Am Coll Cardiol. 2003;42(7):1199-205. Epub 2003/10/03. |
| 268. | Tsimikas S, Mallat Z, Talmud PJ, Kastelein JJP, Wareham NJ, Sandhu MS, et al. Oxidation-specific biomarkers, lipoprotein(a), and risk of fatal and nonfatal coronary events. J Am Coll Cardiol. 2010;56(12):946-55. |
| 269. | Tsimikas S, Willeit P, Willeit J, Santer P, Mayr M, Xu Q, et al. Oxidation-specific biomarkers, prospective 15-year cardiovascular and stroke outcomes, and net reclassification of cardiovascular events. J Am Coll Cardiol. 2012;60(21):2218-29. |
| 270. | Tunstall-Pedoe H. The Dundee coronary risk-disk for management of change in risk factors. BMJ. 1991;303(6805):744-7. Epub 1991/09/28. |
| 271. | Tunstall-Pedoe H, Woodward M, estimation Sgor. By neglecting deprivation, cardiovascular risk scoring will exacerbate social gradients in disease. Heart. 2006;92(3):307-10. |
| 272. | Ulmer H, Kollerits B, Kelleher C, Diem G, Concin H. Predictive accuracy of the SCORE risk function for cardiovascular disease in clinical practice: a prospective evaluation of 44 649 Austrian men and women. Eur J Cardiovasc Prev Rehabil. 2005;12(5):433-41. |
| 273. | Vaidya D, Yanek LR, Moy TF, Pearson TA, Becker LC, Becker DM. Incidence of coronary artery disease in siblings of patients with premature coronary artery disease: 10 years of follow-up. Am J Cardiol. 2007;100(9):1410-5. |
| 274. | van der Heijden AAWA, Ortegon MM, Niessen LW, Nijpels G, Dekker JM. Prediction of coronary heart disease risk in a general, pre-diabetic, and diabetic population during 10 years of follow-up: accuracy of the Framingham, SCORE, and UKPDS risk functions: The Hoorn Study. Diabetes Care. 2009;32(11):2094-8. |
| 275. | van Dis I, Kromhout D, Geleijnse JM, Boer JMA, Verschuren WMM. Evaluation of cardiovascular risk predicted by different SCORE equations: the Netherlands as an example. Eur J Cardiovasc Prev Rehabil. 2010;17(2):244-9. |
| 276. | Veeranna V, Zalawadiya SK, Niraj A, Pradhan J, Ference B, Burack RC, et al. Homocysteine and reclassification of cardiovascular disease risk. J Am Coll Cardiol. 2011;58(10):1025-33. |
| 277. | Venskutonyte L, Ryden L, Nilsson G, Ohrvik J. Mortality prediction in the elderly by an easily measured metabolic index. Diab Vasc Dis Res. 2012;9(3):226-33. Epub 2012/01/27. |
| 278. | Vergnaud AC, Bertrais S, Galan P, Hercberg S, Czernichow S. Ten-year risk prediction in French men using the Framingham coronary score: results from the national SU.VI.MAX cohort. Prev Med. 2008;47(1):61-5. |
| 279. | Verwoert GC, Elias-Smale SE, Rizopoulos D, Koller MT, Steyerberg EW, Hofman A, et al. Does aortic stiffness improve the prediction of coronary heart disease in elderly? The Rotterdam Study. J Hum Hypertens. 2012;26(1):28-34. |
| 280. | Villines TC, Taylor AJ. Multi-ethnic study of atherosclerosis arterial age versus framingham 10-year or lifetime cardiovascular risk. Am J Cardiol. 2012;110(11):1627-30. |
| 281. | Vlismas K, Panagiotakos DB, Pitsavos C, Chrysohoou C, Skoumas Y, Stavrinos V, et al. The role of dietary and socioeconomic status assessment on the predictive ability of the HellenicSCORE. Hellenic J Cardiol. 2011;52(5):391-8. |
| 282. | Voko Z, Hollander M, Koudstaal PJ, Hofman A, Breteler MMB. How do American stroke risk functions perform in a Western European population? Neuroepidemiology. 2004;23(5):247-53. |
| 283. | Voss R, Cullen P, Schulte H, Assmann G. Prediction of risk of coronary events in middle-aged men in the Prospective Cardiovascular Munster Study (PROCAM) using neural networks. Int J Epidemiol. 2002;31(6):1253-62; discussion 62-64. Epub 2003/01/24. |
| 284. | Wang TJ, Gona P, Larson MG, Tofler GH, Levy D, Newton-Cheh C, et al. Multiple biomarkers for the prediction of first major cardiovascular events and death. N Engl J Med. 2006;355(25):2631-9. |
| 285. | Wang Z, Hoy WE. Is the Framingham coronary heart disease absolute risk function applicable to Aboriginal people? Med J Aust. 2005;182(2):66-9. Epub 2005/01/18. |
| 286. | Wannamethee SG, Shaper AG, Lennon L, Morris RW. Metabolic syndrome vs Framingham Risk Score for prediction of coronary heart disease, stroke, and type 2 diabetes mellitus. Arch Intern Med. 2005;165(22):2644-50. |
| 287. | Weiner DE, Tighiouart H, Griffith JL, Elsayed E, Levey AS, Salem DN, et al. Kidney disease, Framingham risk scores, and cardiac and mortality outcomes. Am J Med. 2007;120(6):552.e1-8. |
| 288. | Wilson PW, Castelli WP, Kannel WB. Coronary risk prediction in adults (the Framingham Heart Study). Am J Cardiol. 1987;59(14):91G-4G. Epub 1987/05/29. |
| 289. | Wilson PW, D'Agostino RB, Levy D, Belanger AM, Silbershatz H, Kannel WB. Prediction of coronary heart disease using risk factor categories. Circulation. 1998;97(18):1837-47. Epub 1998/05/29. |
| 290. | Wilson PWF, Nam B-H, Pencina M, D'Agostino RB, Sr., Benjamin EJ, O'Donnell CJ. C-reactive protein and risk of cardiovascular disease in men and women from the Framingham Heart Study. Arch Intern Med. 2005;165(21):2473-8. |
| 291. | Wolf PA, D'Agostino RB, Belanger AJ, Kannel WB. Probability of stroke: a risk profile from the Framingham Study. Stroke. 1991;22(3):312-8. Epub 1991/03/01. |
| 292. | Woodward M, Brindle P, Tunstall-Pedoe H, estimation Sgor. Adding social deprivation and family history to cardiovascular risk assessment: the ASSIGN score from the Scottish Heart Health Extended Cohort (SHHEC). Heart. 2007;93(2):172-6. |
| 293. | Woodward M, Tunstall-Pedoe H, Batty GD, Tavendale R, Hu FB, Czernichow S. The prognostic value of adipose tissue fatty acids for incident cardiovascular disease: results from 3944 subjects in the Scottish Heart Health Extended Cohort Study. Eur Heart J. 2011;32(11):1416-23. |
| 294. | Woodward M, Tunstall-Pedoe H, Rumley A, Lowe GDO. Does fibrinogen add to prediction of cardiovascular disease? Results from the Scottish Heart Health Extended Cohort Study. Br J Haematol. 2009;146(4):442-6. |
| 295. | Woodward M, Welsh P, Rumley A, Tunstall-Pedoe H, Lowe GDO. Do inflammatory biomarkers add to the discrimination of cardiovascular disease after allowing for social deprivation? Results from a 10-year cohort study in Glasgow, Scotland. Eur Heart J. 2010;31(21):2669-75. |
| 296. | Wormser D, Kaptoge S, Di Angelantonio E, Wood AM, Pennells L, Thompson A, et al. Separate and combined associations of body-mass index and abdominal adiposity with cardiovascular disease: Collaborative analysis of 58 prospective studies. The Lancet. 2011;377(9771):1085-95. |
| 297. | Wu Y, Liu X, Li X, Li Y, Zhao L, Chen Z, et al. Estimation of 10-year risk of fatal and nonfatal ischemic cardiovascular diseases in Chinese adults. Circulation. 2006;114(21):2217-25. |
| 298. | Wu Y, Zhang L, Yuan X, Wu Y, Yi D. Quantifying links between stroke and risk factors: a study on individual health risk appraisal of stroke in a community of Chongqing. Neurol Sci. 2011;32(2):211-9. |
| 299. | Xie W, Liang L, Zhao L, Shi P, Yang Y, Xie G, et al. Combination of carotid intima-media thickness and plaque for better predicting risk of ischaemic cardiovascular events. Heart. 2011;97(16):1326-31. |
| 300. | Yip YB, Wong TKS, Chung JWY, Ko SKK, Sit JWH, Chan TMF. Cardiovascular disease: application of a composite risk index from the Telehealth System in a district community. Public Health Nurs. 2004;21(6):524-32. |
| 301. | Zhang X-F, Attia J, D'Este C, Yu X-H, Wu X-G. A risk score predicted coronary heart disease and stroke in a Chinese cohort. J Clin Epidemiol. 2005;58(9):951-8. |
| 302. | Zomer E, Owen A, Magliano DJ, Liew D, Reid C. Validation of two Framingham cardiovascular risk prediction algorithms in an Australian population: the 'old' versus the 'new' Framingham equation. Eur J Cardiovasc Prev Rehabil. 2011;18(1):115-20. |
